# Caspase-8 is required for HSV-1-induced apoptosis and promotes effective viral particle release via autophagy inhibition

**DOI:** 10.1038/s41418-022-01084-y

**Published:** 2022-11-24

**Authors:** Francesca Marino-Merlo, Anusha Klett, Emanuela Papaianni, Selene Francesca Anna Drago, Beatrice Macchi, María Gabriela Rincón, Federica Andreola, Annalucia Serafino, Sandro Grelli, Antonio Mastino, Christoph Borner

**Affiliations:** 1 Department of Chemical, Biological, Pharmaceutical, and Environmental Sciences, University of Messina, 98166 Messina, Italy; 2 Institute of Molecular Medicine and Cell Research, Faculty of Medicine, University of Freiburg, 79104 Freiburg, Germany; 3 Spemann Graduate School of Biology and Medicine (SGBM), University of Freiburg, 79104 Freiburg, Germany; 4 Faculty of Biology, University of Freiburg, 79104 Freiburg, Germany; 5 Department of Chemical Science and Technology, University of Rome “Tor Vergata”, 00133 Rome, Italy; 6 The Institute of Translational Pharmacology, CNR, 00133 Rome, Italy; 7 Department of Experimental Medicine, University of Rome “Tor Vergata”, 00133 Rome, Italy

**Keywords:** Macroautophagy, Immune cell death, Immunopathogenesis

## Abstract

Regulated cell death (RCD) plays an important role in the progression of viral replication and particle release in cells infected by herpes simplex virus-1 (HSV-1). However, the kind of RCD (apoptosis, necroptosis, others) and the resulting cytopathic effect of HSV-1 depends on the cell type and the species. In this study, we further investigated the molecular mechanisms of apoptosis induced by HSV-1. Although a role of caspase-8 has previously been suggested, we now clearly show that caspase-8 is required for HSV-1-induced apoptosis in a FADD-/death receptor-independent manner in both mouse embryo fibroblasts (MEF) and human monocytes (U937). While wild-type (wt) MEFs and U937 cells exhibited increased caspase-8 and caspase-3 activation and apoptosis after HSV-1 infection, respective caspase-8-deficient (caspase-8−/−) cells were largely impeded in any of these effects. Unexpectedly, caspase-8−/− MEF and U937 cells also showed less virus particle release associated with increased autophagy as evidenced by higher Beclin-1 and lower p62/SQSTM1 levels and increased LC3-I to LC3-II conversion. Confocal and electron microscopy revealed that HSV-1 stimulated a strong perinuclear multivesicular body response, resembling increased autophagy in caspase-8−/− cells, entrapping virions in cellular endosomes. Pharmacological inhibition of autophagy by wortmannin restored the ability of caspase-8−/− cells to release viral particles in similar amounts as in wt cells. Altogether our results support a non-canonical role of caspase-8 in both HSV-1-induced apoptosis and viral particle release through autophagic regulation.

## Introduction

Regulated cell death (RCD) by apoptosis [1] is a crucial player of anti-viral immune responses [2, 3]. However, different viruses and their hosts co-evolved complex mechanisms aimed to evade or at least perturb the apoptosis of infected cells [3, 4]. Intriguingly, human herpes simplex virus (HSV)-1 and HSV-2 are provided with subtle and complex RCD-modulating strategies that could be paradigmatic in this respect [5–7]. In fact, the productive infection of fully permissive cells with HSV infection is characterized by an efficient counteraction of the apoptotic response involving numerous viral genes [8], before cells ultimately die by a form of lytic cell death. HSV viral gene products with anti-apoptotic activity include proteins of the IE genes [9, 10], the US3 protein [11–13], glycoprotein gD [14, 15], the gJ protein [14, 16] and the latency-associated transcript [17–19]. Interestingly, however, ICP0 and ICP27 have been identified as pro-apoptotic HSV-1 proteins [20, 21], implying that HSV and their natural hosts retained during evolution their ability to trigger apoptotic RCD in response to infection. In fact, in human cells such as monocytes, dendritic cells or lymphocytes, which can be efficiently infected by HSV but do not optimally sustain virus replication, or in mouse cells, such as mouse embryonic fibroblasts (MEFs), infection is associated with apoptosis as an exclusive cytopathic effect [22–28]. Mechanisms controlling apoptosis and virus replication in cells sensitive to HSV-induced RCD have not been fully elucidated. A recent study by our laboratories revealed that the BH3-only protein Puma is a crucial mediator of HSV-1-induced Bax/Bak activation and consequent apoptotic RCD and that death receptors, known to induce caspase-8 activation, are not involved in this process [24, 28]. Intriguingly, however, results from other authors suggested that caspase-8 could be involved in apoptosis triggered by HSV [26, 29–31].

In order to shed more light on the apparent contradictory data obtained by us and others, we further elucidated the role of caspase-8 in HSV-1 induced apoptosis in caspase-8-deficient mouse embryo fibroblasts (MEFs) and U937 human monocytes. We confirm that caspase-8 is crucial for HSV-1-induced apoptosis and its activation occurs in a non-canonical, death receptor/FADD-independent manner. In addition, we found that caspase-8 activation contributes to efficient HSV-1 particle release by restraining autophagy and thereby preventing the trapping of viral particles in autophagic vesicles.

## Results

### Caspase-8 is required for HSV-1 induced caspase-3 activation and apoptosis

**Fig. 1:**
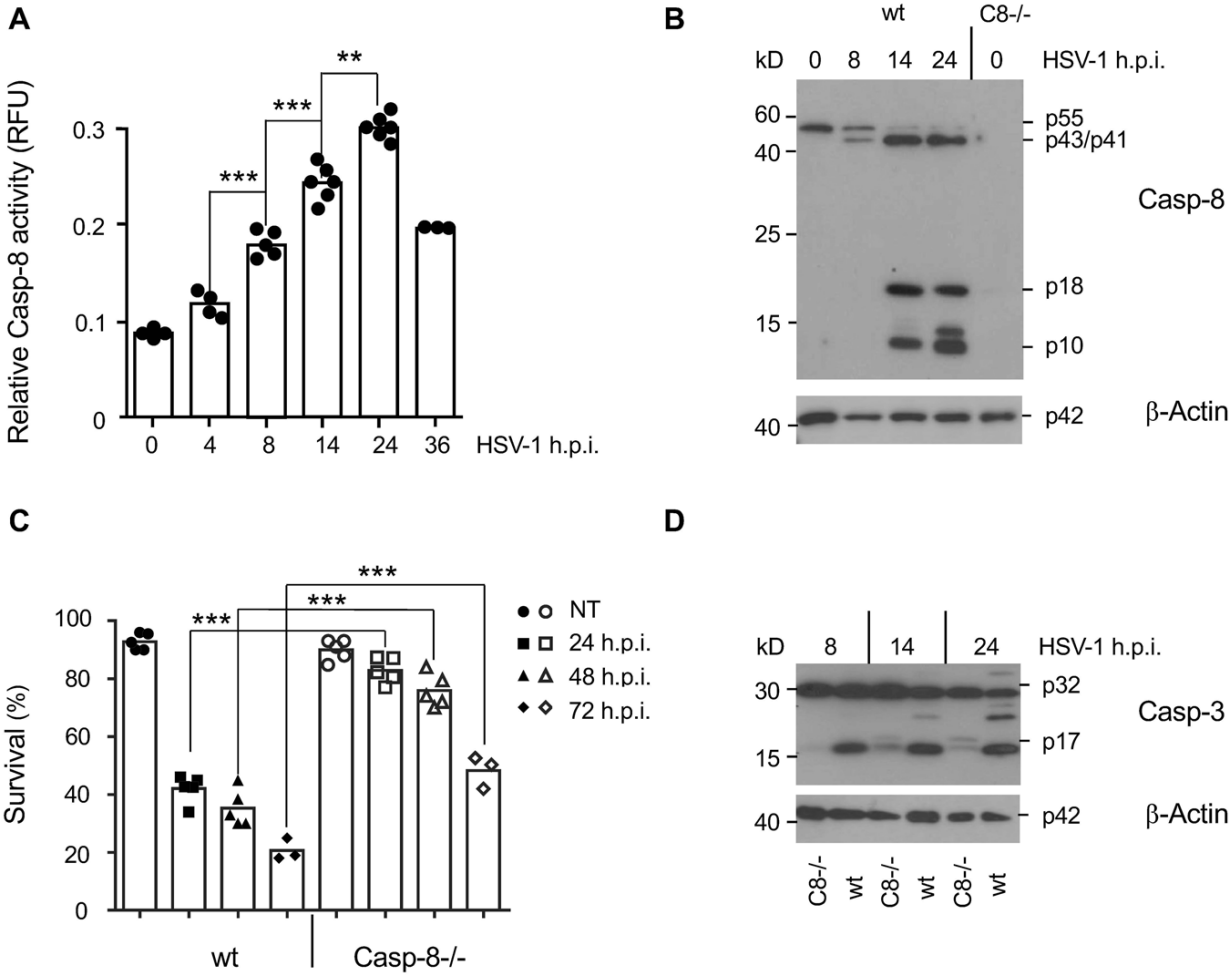
Caspase-8 is activated after HSV-1 infection and required for HSV-1-induced caspase-3 activation and apoptosis in MEFs. **A** Caspase-8 (IETDase) activity assay of total extracts of wild-type (wt) MEFs infected with 10 m.o.i. of HSV-1 for 0 to 36 h. Results are presented as relative fluorescence units (RFU). **B** Anti-caspase-8 western blot analysis of total extracts of MEFs infected with HSV-1 for 0, 8, 14 and 24 h, showing the inactive p55 caspase-8 pro-form and the cleaved, active p43/p41 and p18/p10 forms. The right line shows an extract from caspase-8−/− MEFs. **C** Percentage of FITC-Annexin-V/7-AAD-negative (surviving) wt and caspase-8−/− (Casp-8−/−) MEFs infected with HSV-1 for 0 (NT), 24, 48 and 72 h. **D** Anti-caspase-3 western blot analysis of total extracts of wt and caspase-8−/− MEFs infected with HSV-1 for 8, 14 and 24 h showing the inactive p32 caspase-3 pro-form and the cleaved, active p17 form. Data in **A** and **C** represent the means of 3–6 independent experiments ± SD. Statistical evaluation by one-way ANOVA: **p < 0.01; ***p < 0.001. In **B** and **D**, β-actin served as a loading control.

**Fig. 2:**
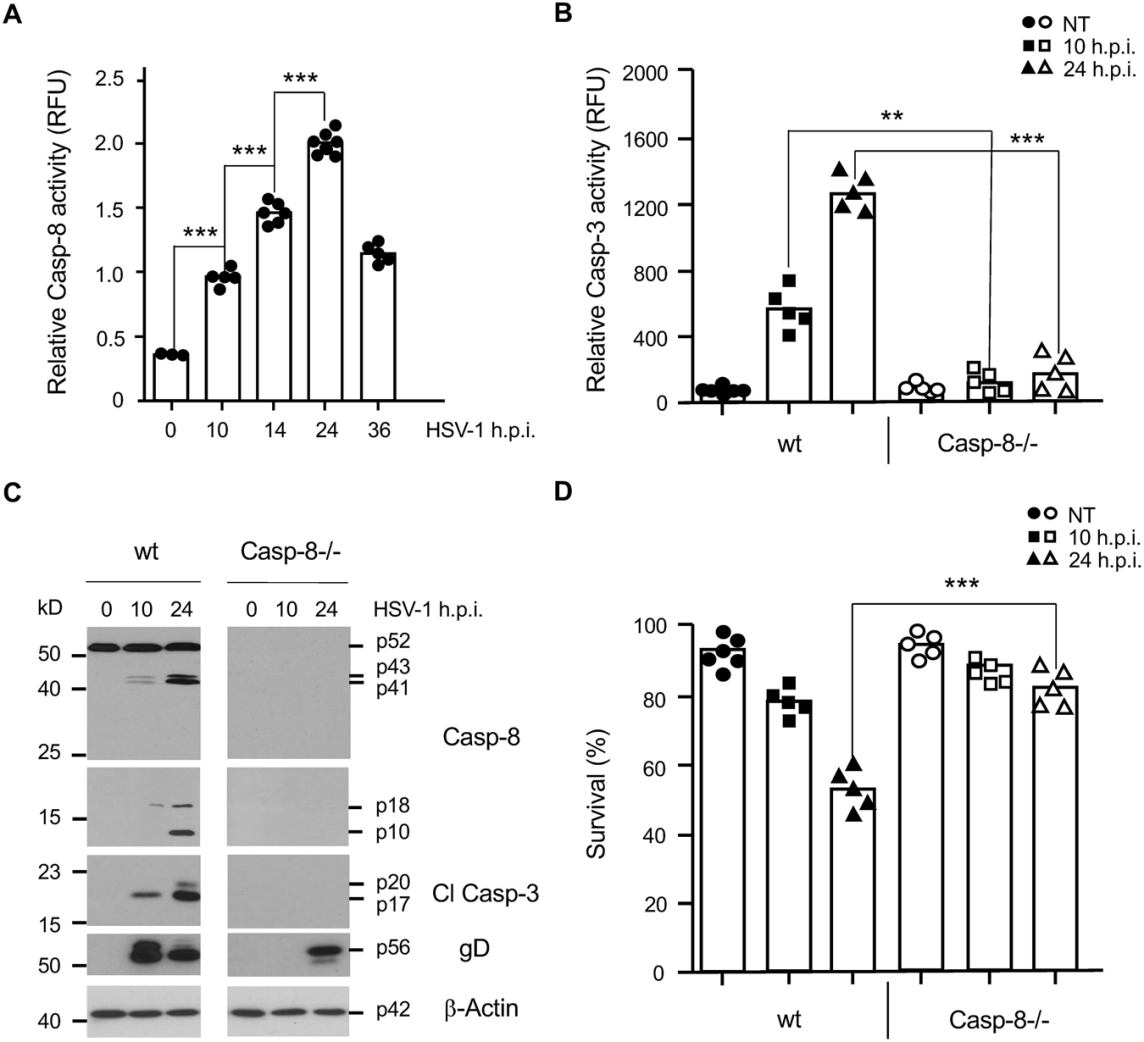
Caspase-8 is activated after HSV-1 infection and required for HSV-1-induced caspase-3 activation and apoptosis in U937 cells. **A** Caspase-8 (IETDase) activity assay of total extracts of wt U937 cells infected with 50 m.o.i. of HSV-1 for 0 to 36 h. **B** Caspase-3 (DEVDase) activity assay on total extracts of wt and caspase-8−/− (Casp-8−/−) U937 cells infected with HSV-1 for 0 (NT), 10 and 24 h. **C** Anti-caspase-8, -caspase-3 and -gD western blot analyses of total extracts of wt and caspase-8−/− U937 cells infected with HSV-1 for 0, 10 and 24 h showing the inactive p55 pro-form and the active p43/p41 and p18/p10 caspase-8 fragments, the active p20/p17 caspase-3 (Cl Casp-3) fragments and the expression of the viral gD protein. β-Actin served as a loading control. **D** Percentage of FITC-Annexin-V/7-AAD-negative (surviving) wt and caspase-8−/− (Casp-8−/−) U937 cells infected with HSV-1 for 0 (NT), 10 and 24 h. Data in **A**, **B** and **D** represent the means of 3–7 independent experiments ± SD. Statistical evaluation by one-way ANOVA: **p < 0.01, ***p < 0.001.

Using a fluorogenic IETD-AFC assay, we found that infection of MEFs with 10 multiplicity of infection (m.o.i.) of HSV-1 gradually increased caspase-8 activity in total cellular extracts up to 24 h post infection (h.p.i.) after which the activity declined (Fig. 1A). This was accompanied by the processing of pro-caspase-8 into its active p43/p41 and p18/p10 forms as evidenced by anti-caspase-8 immunoblot analysis (Fig. 1B). In addition, caspase-3 was processed (Fig. 1D) and the cells underwent apoptosis as determined by FITC-Annexin-V/7-AAD staining (Fig. 1C). Similarly, human U937 cells infected with 50 m.o.i. of HSV-1 showed increased caspase-8 (Fig. 2A) and caspase-3 activity (Fig. 2B), caspase-8 and -3 processing into the active forms (Fig. 2C) and apoptosis induction (Fig. 2D). Next, we studied if caspase-8 was required for HSV-1-induced caspase-3 activation and apoptosis in MEFs and U937 cells. For that purpose, we used MEFs isolated from caspase-8−/− mice and generated U937 cells lacking the caspase-8 gene by CRISPR/Cas9. Both caspase-8−/− MEFs and U937 cells exhibited diminished caspase-3 processing (Figs. 1D and 2C) and activity (Fig. 2B) as well as apoptosis induction (Figs. 1C and  2D) after HSV-1 infection. These data indicate that HSV-1-induced apoptosis depends, at least in part, on a caspase-8- and caspase-3-mediated signalling pathway.

### Caspase-8 activation and apoptosis induced by HSV-1 occur independently of FADD

**Fig. 3:**
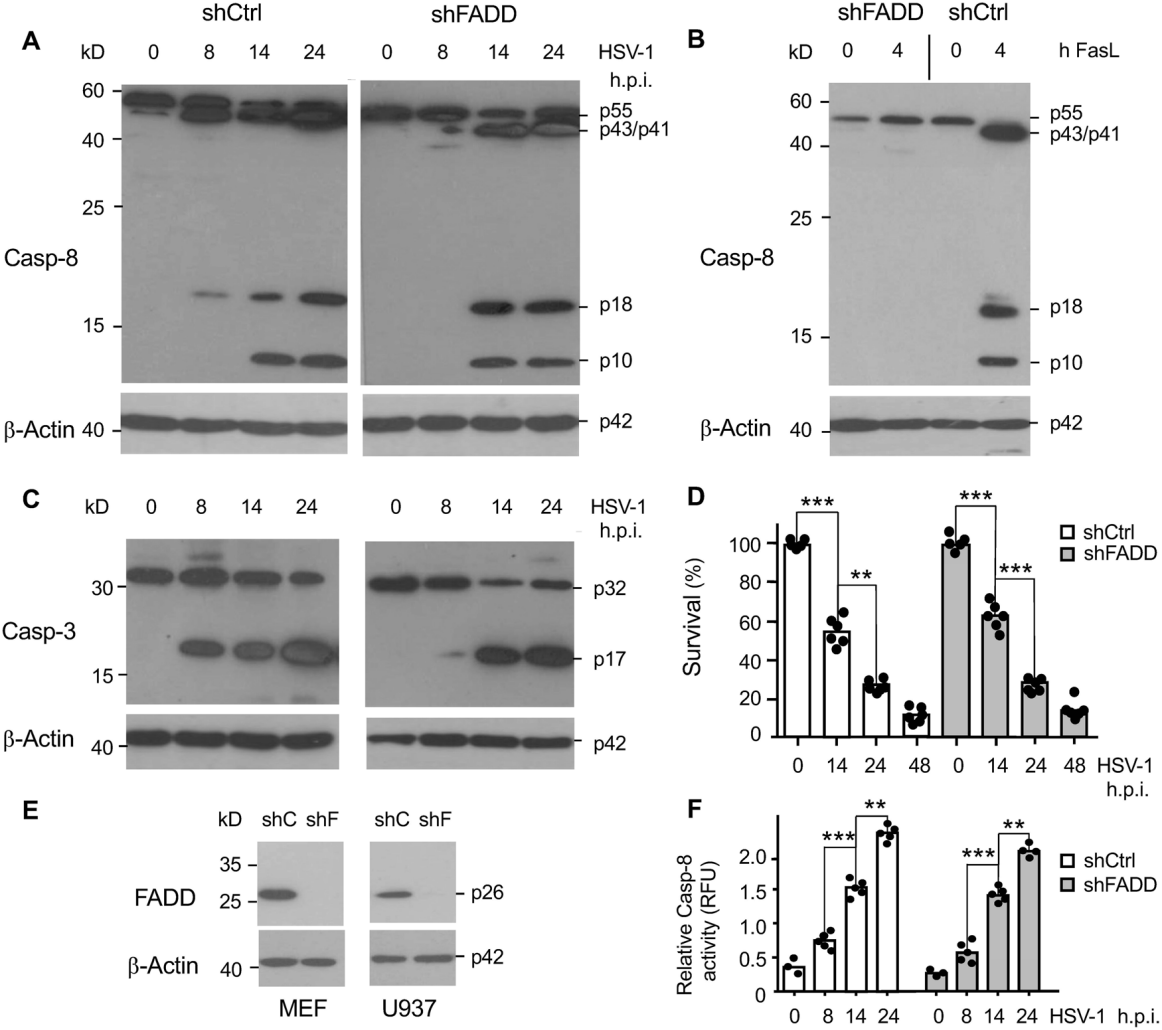
HSV-1-induced apoptosis of MEFs does not require the caspase-8 adaptor/activator FADD. **A, B** Anti-caspase-8 and **C** anti-caspase-3 western blot analysis of total extracts of MEFs either expressing a scrambled shRNA (shCtrl) or a shRNA against FADD (shFADD), either mock-infected (0 h) or infected with HSV-1 for 8, 14 and 24 h (**A**, **C**) or treated with 50 ng/ml FasL for 4 h (**B**). The extent and kinetic of caspase-8 processing to the active p43/p41 and p18/p10 fragments and the processing of caspase-3 to the active p17 fragment are similar in shCtrl and shFADD MEFs. β-Actin served as a loading control. **D** Percentage of FITC-Annexin-V/7-AAD-negative (surviving) shCtrl and shFADD MEFs, either mock-infected (0 h) or infected with HSV-1 for 14, 24 and 48 h showing that the kinetics of HSV-1-induced apoptosis is not largely affected by the absence of FADD. **E** Anti-FADD western blot analysis showing the efficient knockdown of FADD in MEFs and U937 cells stably expressing FADD shRNAs (shF). As a control, a scrambled shRNA was transduced (shC). β-actin served as a loading control. **F** Caspase-8 (IETDase) activity assay of total extracts of shCtrl and shFADD U937 cells infected with 50 m.o.i. of HSV-1 for 0, 8, 14 and 24 h showing a similar extent and kinetic of caspase-8 activation between the two cell lines. Data in **D** and **F** represent the means of 3–6 independent experiments ± SD. Statistical evaluation by one-way ANOVA: **p < 0.01; ***p < 0.001.

Canonical caspase-8 activation occurs due to its recruitment to the death-inducing signalling complex (DISC) after FasL, TNF or TRAIL-induced death receptor stimulation via the adaptor protein FADD. We [28] and others [24] have previously shown that HSV-1-induced apoptosis does not require FasL, TNF, TRAIL or their receptors. This however does not exclude the recruitment of caspase-8 to another FADD-containing platform than the DISC for its activation. We therefore knocked down the crucial adaptor FADD by shRNA in MEF and U937 cells (shFADD) (Fig. 3E). In HSV-1-infected shFADD MEFs, the active p43/p41 and p18/10 caspase-8 fragments (Fig. 3A) and the active p17 caspase-3 fragment (Fig. 3C) were still formed with a similar time kinetic as in sh scrambled control (shCtrl) cells. Also, the extent of apoptosis in response to HSV-1 infection was not any different in these cells when FADD expression was ablated (Fig. 3D). By contrast, caspase-8 processing was effectively blocked in shFADD MEFs treated with FasL (Fig. 3B) indicating an effective downregulation of FADD expression (Fig. 3E). Similarly, U937 cells lacking FADD expression exhibited a caspase-8 activation kinetic indistinguishable from shCtrl cells (Fig. 3F). These data indicate that HSV-1-induced caspase-8 and caspase-3 activation as well as apoptosis mainly occur independently of the adaptor FADD and therefore involve an activation platform different from the canonical DISC.

### Caspase-8 is not only required for HSV-1-induced apoptosis but also for the effective release of viral particles

**Fig. 4:**
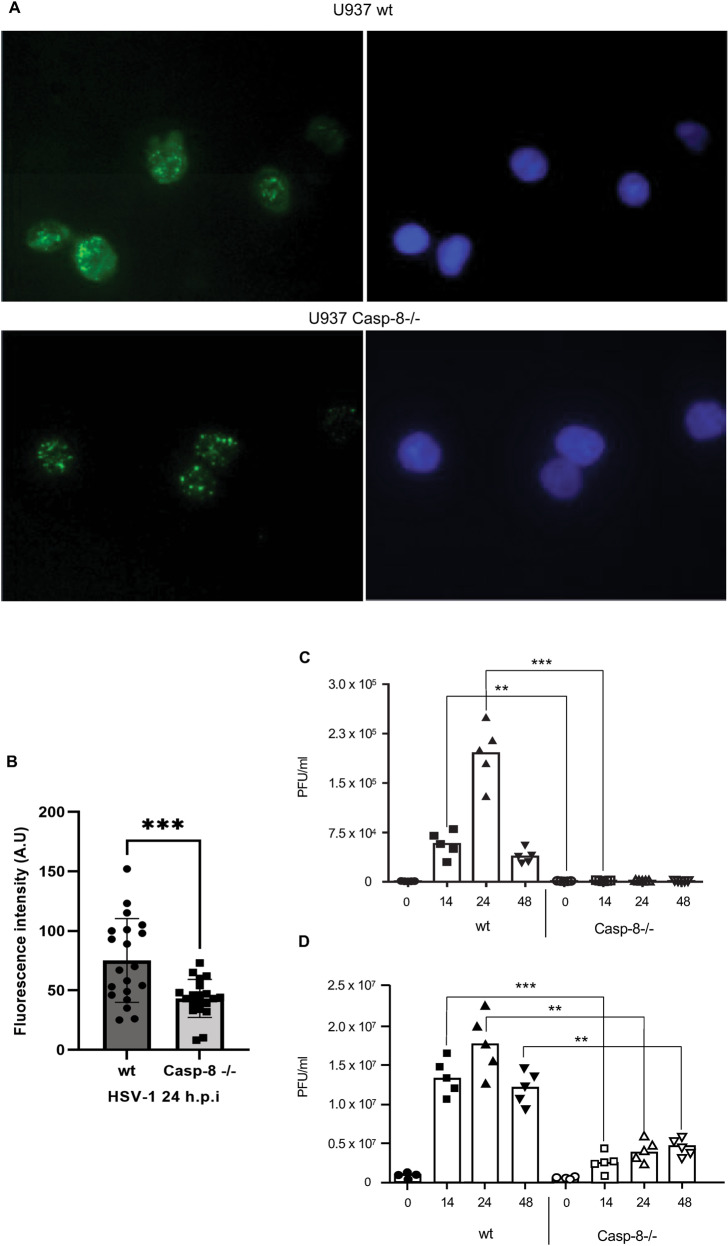
Caspase-8 facilitates effective cellular release of HSV-1 virions. **A** Anti-gD immunofluorescence analysis of wt and caspase-8−/− U937 cells infected with 50 m.o.i. of HSV-1 for 16 h. Nuclei were stained with Hoechst 33342. **B** Quantitation of anti-gD fluorescence intensity in relation to Hoechst 33342 of 20 individual cells from 3 independent experiments ± SD using ImageJ version 1.53k. Statistical evaluation is by two-sample Student’s t-test, ***p < 0.001. Note that caspase-8−/− cells exhibit an approximatively twofold lower gD immunostaining. **C**, **D** Quantitation of virus titres in the supernatant of wt and caspase-8−/− MEFs (B) or U937 cells (**C**) infected with HSV-1 for 0, 14, 24 and 48 h, as determined by plaque assay on Vero cells. Viral titres are shown as plaque forming unit (PFU) per ml. Caspase-8−/− cells secrete less HSV-1 particles than wt cells. Data represent the means of 3–5 independent experiments ± SD. Statistical evaluation by one-way ANOVA: **p < 0.01, ***p < 0.001.

We next wanted to know if the resistance of caspase-8−/− cells to HSV-1-induced apoptosis also affected virus replication and particle release. For that purpose, we monitored the expression of the late viral protein gD by immunofluorescence and determined the viral titre in the cellular supernatants of HSV-1-infected wt and caspase-8−/− MEFs and U937 cells by plaque assays. Anti-gD immunofluorescence revealed an approximatively twofold lower expression of the viral gD protein in caspase-8−/− as compared to wt U937 cells after 24 h of HSV-1 infection (Figs.  2C and 4A, B). Consistent with this notion, the viral titres were markedly diminished in both caspase-8−/− U937 cells and MEFs as compared to their wt counterparts (Fig. 4C, D) indicating that caspase-8 was crucial for completing the virus reproduction cycle allowing the proper egress of infectious virus particles after HSV-1 infection.

### Caspase-8 facilitates Beclin-1 downregulation and restrains autophagy in response to HSV-1 infection

**Fig. 5:**
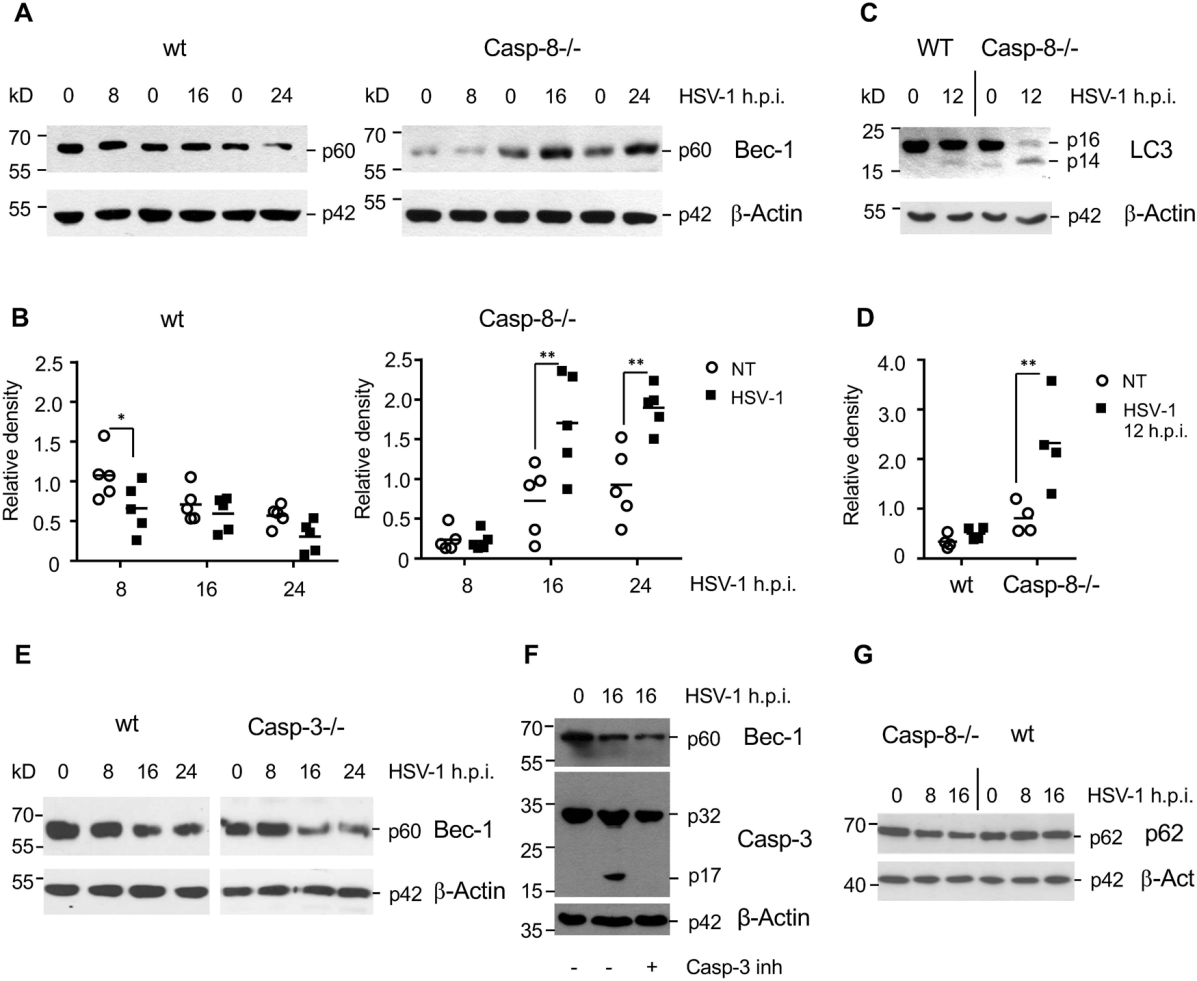
Caspase-8 restrains autophagy after HSV-1 infection in MEFs. **A** Anti-Beclin-1 western blot analysis of total extracts of wt and caspase-8−/− MEFs, either mock-infected (0 h) or infected with 10 m.o.i. of HSV-1 for 8, 16 and 24 h. **B** Quantitation of the Beclin-1 protein bands shown in (**A**) by densitometric analysis of scanned films using the ImageJ software. HSV-1-infected caspase-8−/− cells express higher levels of Beclin-1 than wt cells. **C** Anti-LC3 western blot analyses of wt and caspase-8−/− MEFs either mock-infected (0 h, NT) or infected with HSV-1 for 12 h. **D** Quantitation of the respective LC3-II protein bands shown in C. Caspase-8−/− cells exhibit increased autophagy after HSV-1 infection. **E, F** Anti-Beclin-1 western blot analysis of total extracts of wt and caspase-3−/− MEFs (**E**) or wt MEFs treated with 100 μM of the caspase-3 inhibitor Ac-DEVD-CMK (**F**), either mock-infected (0 h) or infected with 10 m.o.i. of HSV-1 for 8, 16 and 24 h. Beclin-1 is degraded with similar kinetics in the presence or absence of caspase-3 expression. **G** Anti-p62/SQSTM1 western blot analysis of total extracts of wt and caspase-8−/− MEFs either mock-infected (0 h) or infected with 10 m.o.i. of HSV-1 for 8 and 16 h. p62 is slightly more degraded in HSV-1-infected Casp-8−/− than wt cells suggesting increased autophagy completion in the former. β-Actin served as a loading control in **A**, **C**, **E**, **F** and **G**. Data in **B** and **D** are depicted as relative densities and represent the means of 3–5 independent experiments ± SD. Statistical evaluation by one-way ANOVA: *p < 0.05, **p < 0.01, ***p < 0.001.

**Fig. 6:**
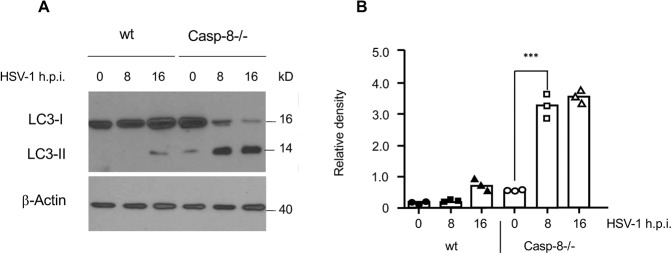
Caspase-8 restrains autophagy after HSV-1 infection in U937 cells. **A** Anti-LC3 western blot analyses of wt and caspase-8−/− U937 cells either mock-infected (0 h) or infected with HSV-1 for 8 and 16 h. β-Actin served as a loading control. **B** Quantitation of the respective LC3-II protein bands shown in **A**. Autophagy is significantly increased in caspase-8−/− U937 as compared to wt cells. Data are depicted as relative densities and represent the means of 3 independent experiments ± SD. Statistical evaluation by one-way ANOVA: ***p < 0.001.

We reasoned that caspase-8 may impact on the release of viral particles by regulating their autophagic uptake. It has been previously shown that the crucial autophagic component Beclin-1 is a direct substrate for caspase-8 [32–34]. We therefore performed anti-Beclin-1 immunoblot analysis of total extracts from HSV-1-infected wt and caspase-8−/− MEFs. As shown in Fig. 5A, B, while in wt MEFs the protein levels of Beclin-1 diminished within 24 h.p.i., this was not the case in caspase-8−/− cells. Beclin-1 cleavage in response to HSV-1 infection seemed to be a direct effect of caspase-8 activation as it still occurred in caspase-3-deficient MEFs (Fig. 5E) or in MEFs treated with the specific caspase-3 inhibitor (Ac-DEVD-CMK) (Fig. 5F). Consistent with this notion, HSV-1-infected caspase-8−/− cells exhibited a higher conversion of LC3-I to LC3-II than wt counterparts (Fig. 5C, D). In addition, the expression of the cargo receptor p62, which is responsible for facilitating the clearance of ubiquitinated proteins in autophagosomes [35, 36], was somewhat diminished in caspase-8−/− MEFs after 8 and 16 h of HSV-1 infection (Fig. 5G). Our finding was confirmed in U937 cells, which also showed a higher LC3-I to LC3-II conversion in caspase-8−/− than wt cells after HSV-1 infection (Fig. 6A, B). These data indicate that the rate of induction (Beclin-1 stabilization) and completion (p62 degradation) of autophagy in response to HSV-1 infection was enhanced when caspase-8 expression was ablated.

### HSV-1 viral particles co-localize with and are entrapped within autophagic vesicles in caspase-8−/− MEFs

**Fig. 7:**
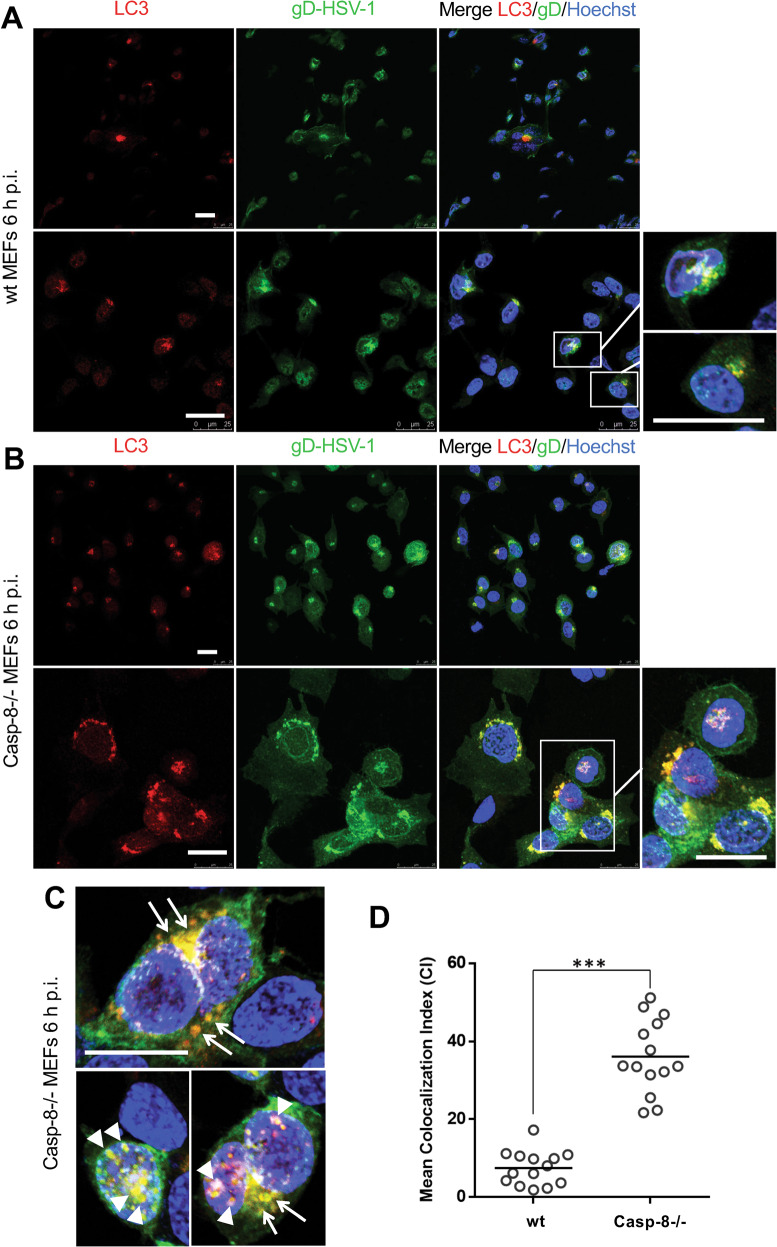
Viral particles co-localize with LC3-positive autophagic vesicles in HSV-1-infected caspase-8−/− MEFs. **A**, **B** Anti-gD (green) and anti-LC3 (red) immunofluorescence analysis by confocal microscopy of wt (**A**) or caspase-8−/− (**B**) MEFs infected with 10 m.o.i. of HSV-1 for 6 h, and counterstained with Hoechst 33342 for nuclei visualization (blue). Images from single confocal sections are shown. The right panels show the merged images of the three fluorophores used, with a magnification in the lower, far right panel. Scale bar: 25 μm. **C** Further magnification of HSV-1-infected caspase-8−/− MEFs showing the colocalization (yellow colour) of viral particles (anti-gD green) with autophagic vesicles (anti-LC3 red) in the perinuclear and nuclear areas. Scale bar: 25 μm. **D** Quantitative analysis of colocalization of LC3 and gD-HSV-1 in wt and caspase-8−/− infected MEFs. Results, reported as mean Colocalization Index (CI) ± SD, were calculated as described in Materials and Methods by analyzing a minimum of 10 fields/sample. Significance (two-sample Student’s t-test) *vs* wt MEFs, ***p < 0.001.

**Fig. 8:**
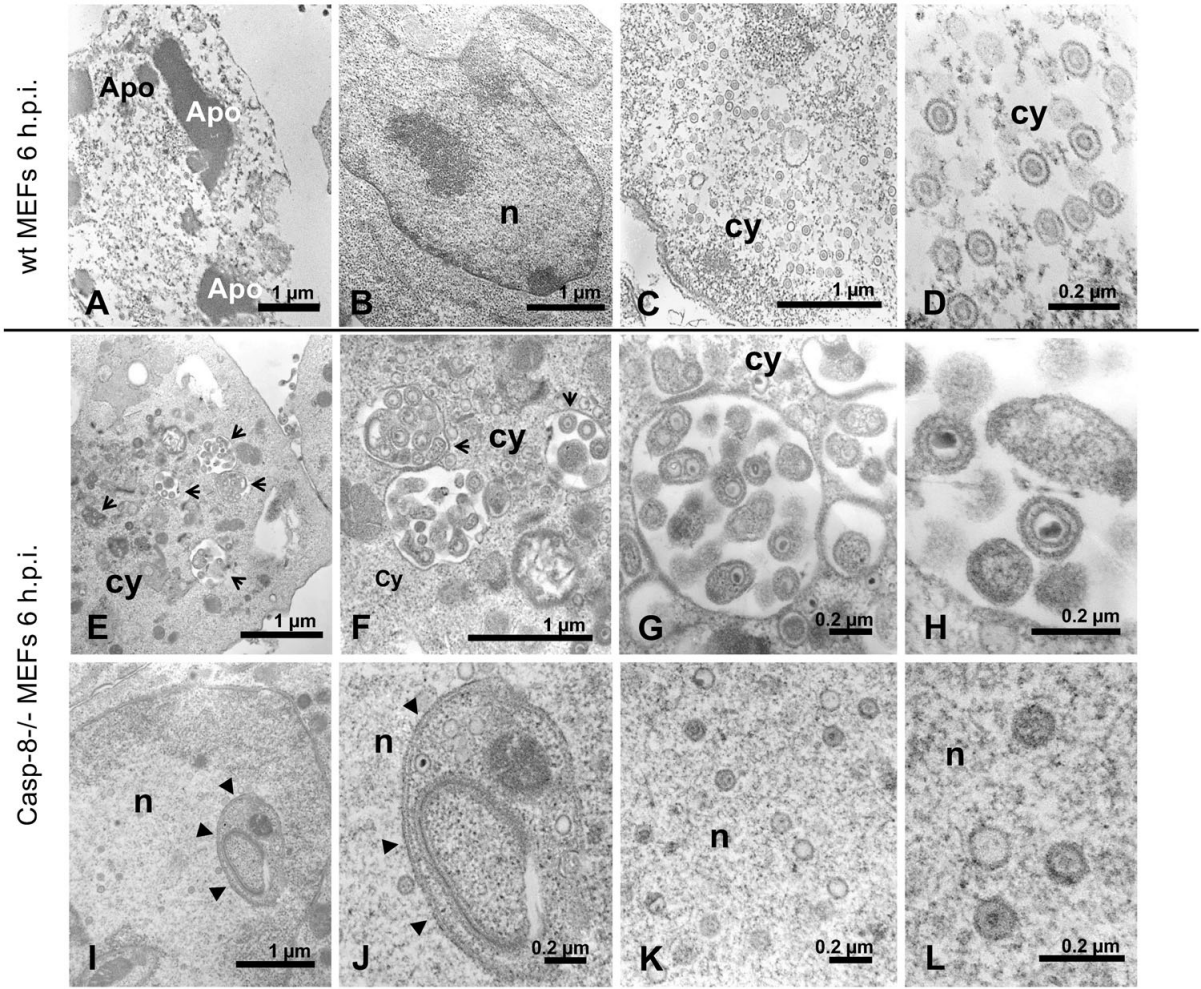
HSV-1 virions are entrapped in cellular membranous structures resembling autophagic vesicles in caspase-8−/− MEFs. Transmission electron microscopy images showing the ultrastructural morphology of wt (**A**–**D**) or caspase-8−/− (**E**–**L**) MEFs infected with 10 m.o.i. of HSV-1 for 6 h. At higher magnifications, the presence of both mature virions and incomplete viral-like particles are well recognized in the cytoplasm of wt MEFs (**C**, **D**). HSV-1 infected caspase-8−/− MEFs show numerous cytoplasmic (**E**–**H**, arrows) and nuclear (**I**–**L**, arrowheads) vesicles in which the numerous virions appear to be entrapped. Apo: apoptotic bodies; n: nucleus; cy: cytoplasm. Scale bars: 1 μm for **A**, **B**, **C**, **E**, **F**, **I**; 200 nm for **D**, **G**, **H**, **J**, **K**, **L**.

**Fig. 9:**
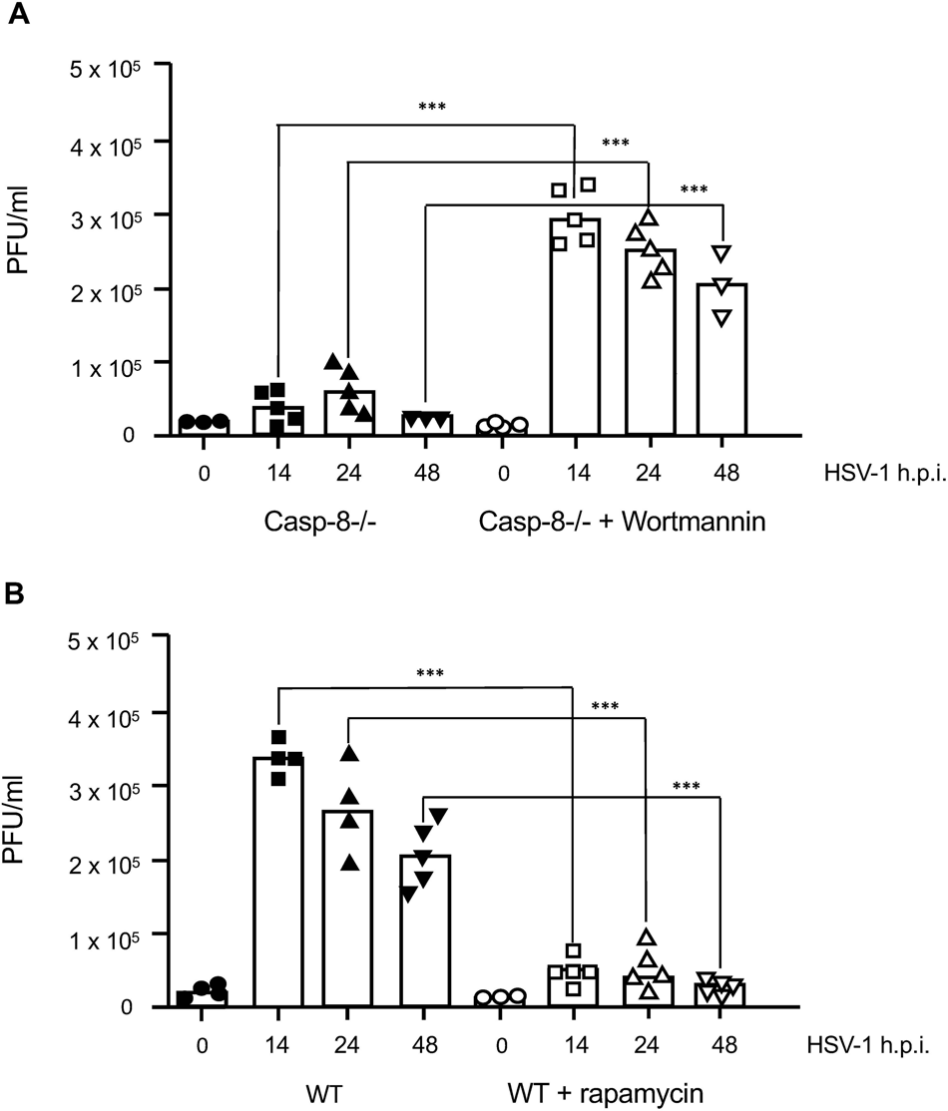
Manipulating autophagy affects the release of virions from HSV-1 infected wt and caspase-8−/− MEFs. **A**, **B** Quantitation of virus titres by plaque assay in the supernatant of wt (**B**) and caspase-8−/− MEFs (**A**) either mock-infected (0 h) or infected with 10 m.o.i. of HSV-1 for 14, 24 and 48 h and either untreated or treated with 0.5 μM wortmannin (**A**) or 5 μM rapamycin 1 h prior to infection (**B**). Viral titres are shown as plaque forming unit (PFU) per ml. HSV-1-infected caspase-8−/− cells secrete more viral particles when autophagy is blocked by wortmannin (**A**). Conversely, HSV-1-infected wt cells release less viral particles when autophagy is activated by rapamycin (**B**). Data represent the means of 3–5 independent experiments ± SD. Statistical evaluation by one-way ANOVA: ***p < 0.001.

Based on our findings, we envisioned that the increased production of autophagic vesicles in caspase-8−/− MEFs may trap viral particles therefore diminishing their release from HSV-1-infected cells. We therefore first studied if the gD protein of HSV-1 viral particles indeed co-localized with autophagic vesicles. For that purpose, we performed anti-gD and LC3 immunofluorescence analysis of HSV-1-infected wt and caspase-8−/− MEFs by confocal microscopy. Consistent with the literature [37] and our data shown in Fig. 5C, D, wt MEFs showed a small number of LC3 puncta reflecting LC3-I to LC3-II conversion at 6 h HSV-1 postinfection (Fig. 7A). These puncta were located almost exclusively in the perinuclear zone of few cells and only partially co-localized with gD-expressing areas (see higher magnification in the lower right panel of Fig. 7A). By contrast, a high number of LC3 puncta were detected in the perinuclear and nuclear region of HSV-1-infected caspase-8−/− MEFs where they co-localized, at least in part with anti-gD immunostaining (Fig. 7B, C). A quantitative analysis of LC3 and HSV-1-gD co-staining revealed a highly significant (*p* = 1.42 × 10^-10^) 4.5-fold increment of the Mean Colocalization Index (CI) in infected caspase-8−/− as compared to wt MEFs (Fig. 7D). These data indicate that viral particles, which are known to egress from the nucleus after virion assembly, could be trapped in LC3-containing autophagic vesicles when caspase-8 expression is ablated. To further investigate the nature and the characteristics of these LC3/gD-positive vesicles in HSV-1-infected caspase-8−/− MEFs, ultrastructural analysis by electron microscopy was performed. As shown in Fig. 8A–D, numerous free HSV-1 virions were distributed throughout the cytoplasm of infected wt cells, and these cells displayed signs of apoptosis including chromatin condensation and nuclear fragmentation (Fig. 8A, Apo). By contrast, ultrastructural analysis of caspase-8−/− MEFs revealed a somewhat different picture. Here, a high proportion of HSV-1 virions were entrapped in cellular membranous structures resembling autophagic vesicles (Fig. 8E–H). These vesicles had a characteristic double membrane, and in some cases, contained other organelles such as mitochondria. Some of the virion-containing autophagic vesicles were also detected within the nucleus (Fig. 8I–L and Fig. 7C, lower panel). Furthermore, both cytoplasmic and nuclear vesicles did not only contain enveloped complete virions, but also defective viral structures, such as empty coats or DNA containing particles without capsids or external coats (Fig. 8K, L). Altogether, our immunofluorescence and electron microscopy data suggest an inhibitory role of caspase-8 on autophagy to prevent HSV-1 virions from being entrapped in perinuclear and cytoplasmic autophagic vesicles and instead be effectively released from infected cells. Consistent with this notion, we found that viral titres were increased in HSV-1-infected caspase-8−/− MEFs when autophagy was inhibited by the PI3K inhibitor wortmannin (Fig. 9A). Conversely, stimulating autophagy with rapamycin in wt MEFs was associated with a diminished cellular release of HSV-1 viral particles (Fig. 9B). Thus, in addition to driving apoptosis, caspase-8 allows effective viral propagation by restraining autophagy.

## Discussion

In this study, we show that caspase-8 is required for HSV-1-induced caspase-3 activation and apoptosis of MEFs and U937 cells. The activation of caspase-8 does not involve its adaptor FADD and hence seems to occur in a non-canonical manner. This is consistent with previous reports that HSV-1-induced apoptosis proceeds independently of death receptor activation [24, 28]. The mechanism by which HSV-1 engages caspase-8 is not yet known. Recently, Choi et al. published that active caspase-8 can be detected in mitochondria following lytic reactivation of human herpesvirus 8 [38]. We however find that while Semliki Forest Virus (SFV) recruits and activates caspase-8 on mitochondria as previously published [39], HSV-1 is not able to do so although caspase-8 can translocate to mitochondria in an inactive pro-form later during infection (Fig. S1). Moreover, we also have not obtained evidence for an involvement of p38 MAPK in the activation of caspase-8 as recently suggested [40] (Fig. S2). However, our study lends support to published data that the virus can under certain circumstances express caspase-8 inhibitory proteins such as the RHIM-domain containing R1 proteins ICP10 and ICP6, which block apoptosis in particular cell types such as epithelial cells [29–31]. Why in MEFs and U937 cells these inhibitory proteins do not effectively act, is yet unclear. It however shows that caspase-8 is a major player in apoptosis induction because otherwise HSV-1 would not have created a strategy to oppose it. In addition to caspase-8, we have recently shown that HSV-1-induced apoptosis also required the BH3-only protein Puma by stabilizing the Puma protein and thereby triggering Bax/Bak-induced MOMP [28]. It is possible that after HSV-1 infection of MEFs and U937 cells, two apoptosis signalling pathways are activated in parallel, one implying Puma-mediated MOMP and another one involving caspase-8-mediated caspase-3 activation. Alternatively, caspase-8-mediated cleavage of the BH3-only protein Bid [41] may trigger additional Bax/Bak activation which acts in concert with Puma. Unfortunately, we have not been able to detect any Bid cleavage in response to HSV-1 infection and Bid−/− MEFs are not particularly protected from HSV-1-induced apoptosis. It therefore remains enigmatic how the caspase-8 and Puma apoptotic signalling pathways interact with each other to bring about maximal HSV-1-induced apoptosis.

A novel aspect of our study is that in addition to being crucial for HSV-1-induced apoptosis, caspase-8 also inhibits HSV-1-induced autophagy. This seems to allow newly generated HSV-1 particles to move freely through the cytoplasm to reach the plasma membrane where they can bud off as infectious virions. In caspase-8−/− cells, HSV-1 infection stimulates a strong perinuclear vesicular response resembling autophagy, capable of entrapping virions in autophagosomes which co-localize with the autophagic marker LC3-II. As a consequence, viral titres drop but can be reinstated by blocking autophagy with wortmannin. Conversely, if autophagy is activated by rapamycin in wt cells, virus egress is diminished most likely because the viral particles are entrapped in autophagosomes. We have not yet been able to follow up the fate of HSV-1-containing autophagosomes in caspase-8−/− or rapamycin-treated wt cells but speculate that they may fuse with lysosomes to be degraded. This would explain why HSV-1-gD is sometimes less detectable (Fig. 4A, B) or less expressed (Fig. 2C), in caspase-8−/− as compared to wt cells. The question remains how caspase-8 inhibits autophagy in HSV-1-infected cells. Pro-caspase-8 has been shown to localize with autophagosomes via the ATG8/LC3-interacting molecule p62/SQSTM1 [42]. p62 serves as an adaptor which binds poly-ubiquitinated (polyU) protein aggregates and targets them for sequestration at the site of autophagosome formation [35, 36]. It is therefore possible that p62 brings polyU caspase-8 oligomers into close proximity thereby facilitating their activation in a DISC-independent manner [36, 43]. Indeed, we found that p62/SQSTM1 is more degraded in caspase-8−/− as compared to wt cells. (Fig. 5G). After activation caspase-8 may cleave substrates crucial for autophagy initiation and/or maturation. We found that after HSV-1 infection the expression levels of Beclin-1 diminish, and this is ablated in caspase-8−/− but not in caspase-3−/− cells or in cells treated with the caspase-3 inhibitor Ac-DEVD-CMK indicating that caspase-8 directly impinges on autophagy by cleaving ATG proteins. It was indeed reported that Beclin-1, but also ATG5 and ATG3, are caspase-8 substrates in vitro, and that after death receptor activation in several cancer cells, the subsequent downregulation of autophagic flux is partly due to caspase-8-dependent cleavage of these autophagic proteins [32–34, 44, 45]. Recently, Musarra-Pizzo et al. confirmed that during HSV-1 replication, activated caspase-8 cleaves the ATG3 protein to potentially block autophagy and support HSV-1 replication and virus particle release [46]. Another finding showed that the processing of Beclin-1 by initiator and effector caspases generated a C-terminal fragment which possessed increased apoptotic properties [34, 47]. The truncated Beclin-1 fragment was found to localize to mitochondria and facilitate MOMP by interacting with pro-survival Bcl-2 family members [34, 47]. Although not proven yet, Beclin-1 cleavage may be a mechanism by which caspase-8 inhibits both autophagy and triggers apoptosis in a Bid-independent, but Puma-amplifying manner in HSV-1-infected cells.

Although different forms of autophagic-like processes have been observed after HSV-1 infection in different cell lines [37, 48–50], the role of autophagy in viral replication and virion egress has remained enigmatic. In specific experimental models an autophagic response induced by HSV-1 could sustain viral replication [51, 52]. On the other hand, it has been shown that HSV-1 is well equipped to evade autophagy [53–55] and that autophagy-related pathways concur in the degradation of HSV-1 virions and significantly inhibit HSV-1 infection [56, 57]. Our data here support this notion as increased autophagy in the absence of caspase-8 or triggered by rapamycin diminishes the release of HSV-1 particles. Thus, caspase-8 could represent a link between apoptosis and autophagy signalling pathways that have so far been considered as separate anti-viral mechanisms to limit HSV-1 replication and to protect uninfected cells [58]. A pro-host-cell interpretation of our proposed model is that in cells prone to apoptosis in response to HSV-1 infection, such as fibroblasts and monocytes, an efficient cooperation between apoptosis and autophagy governed by caspase-8 could avoid overlapping processes with the same purpose and hence the waste of energy inside the cell. On the other hand, a pro-virus interpretation of the caspase-8-centred model is that under normal conditions HSV-1 hijacks the activity of this cellular protein for its own benefit to evade the perinuclear entrapping, therefore allowing effective cellular egress of viruses. In any case, from a co-evolutionary point of view, such mechanisms could be beneficial in part for the virus and in part for the host, thus ensuring the survival of both the human and the virus species.

In conclusion, the experiments carried out in this study have allowed to gather new information on the key role of caspase-8 in modulating the cross-talk between apoptosis and autophagy and hence the outcome of infections in cells prone to apoptosis when exposed to HSV-1. We suspect that caspase-8 could exert a similar apoptosis/autophagy regulating role in infections caused by viruses other than HSV-1.

## Materials and methods

### Cells, viruses and infection

Wild-type (wt), scrambled control (shCtrl) and FADD knockdown (shFADD), caspase-8−/− and caspase-3−/− mouse embryonic fibroblasts (MEFs) as well as wt U937 cells were obtained and cultured as previously reported [28, 39, 59]. Caspase-8−/− U937 cells were generated by CRISPR-Cas9 as follows: The gRNAs targeting the human caspase-8 locus were designed using the web tool CRISPOR (http://crispor.org). The gRNAs with the highest score and the least potential off-target activities were caccgctcttccgaattaatagac (sense) and aaacgtctattaattcggaagagc (antisense) (Eurofins GeneScan, Freiburg, Germany). The gRNAs were cloned into the lentiviral LentiCRISPRv2 plasmid (#52961, Addgene, Watertown, MA, USA). LentiCRISPRv2 Luciferase (kindly provided by Ulrich Maurer, Freiburg) and LentiCRISPRv2 casp8 plasmids were transfected into HEK 293 T cells to produce lentiviruses which were in turn used to generate U937 Luciferase (wt) and U937 caspase-8−/− cells. The cells were plated onto 96-well plates (0.5 cells per well) to pick single cell clones and the genetic deletion of caspase-8 was confirmed by PCR and western blotting. Two clones #14 and #48 were selected for further experiments. The lentiviral short hairpin RNA (shRNA) to knockdown human FADD (gaccgagctcaagttccta) and a scrambled control shRNA (SHC007 luciferase) were obtained from Sigma-Aldrich (Taufkirchen, Germany). Lentiviruses carrying human FADD shRNA were generated as previously described [39]. A total of 1 × 10^6^ U937 cells were infected with 400 μl shRNA lentiviral supernatants in the presence of 5 μg/ml polybrene (Sigma-Aldrich, Taufkirchen Germany) and centrifuged at 2000 rpm at room temperature for 10 min. 3 h postinfection, the cells were washed with PBS, cultured in full media for at least 18 h, and then selected with puromycin (Sigma-Aldrich, Taufkirchen, Germany) for stable expression of the shRNA construct.

HSV-1 virus strain F, originally obtained from ATCC (Manassas, VA, USA), was propagated, stored and titrated as previously described [60]. A multiplicity of infection (m.o.i.) of 10 plaque forming units (PFU)/cell was used for MEFs while a m.o.i. of 50 PFU/cell was used for U937 cells. MEFs were also infected with the virulent SFV prototype strain L10 at 20 m.o.i, as previously described [39].

### Antibodies and reagents

The following antibodies were used: rabbit polyclonal antibodies against active cleaved caspase-3 (#9661, 1:1000), procaspase-3 (#9662, 1:1000), Beclin-1 (#3738, 1:1000) or LC3B (#2775, 1:1000) (Cell Signalling Technology, Danvers, MA, USA); a rabbit polyclonal antibody against human/mouse caspase-8 (AF1650) (R&D systems, Minneapolis, MN, USA, 1:1000); a rabbit polyclonal antibody against β-actin (ab8227) (Abcam, Cambridge, UK, 1:1000); a mouse monoclonal antibody against HSV-1-gD DL6 (sc-21719) (Santa Cruz Biotechnology, Santa Cruz, CA, USA, 1:1000); a rabbit polyclonal antibody against p62/SQSTM1 (ab91526) (Abcam, Cambridge, UK, 1:1000); a mouse monoclonal antibody against mitochondrial α-V-ATPase (7H10) (Molecular Probes/Thermo Fisher Scientific, Freiburg, Germany); secondary goat anti-rabbit and anti-mouse IgG HRP-conjugated (Santa Cruz Biotechnology, Santa Cruz, CA, USA); secondary fluorescein isothiocyanate (FITC)-conjugated and horseradish peroxidase (HRP)-conjugated anti-mouse IgG antibodies (Chemicon, Merck Life Science S.r.l., Milan, Italy). The mouse monoclonal anti-FADD antibody (clone 7A2, 1:1000) was generously provided by Andreas Strasser, WEHI, Australia. The fluorogenic caspase-8 substrate Ac-IETD-AFC, the caspase-3 substrate Ac-DEVD-AMC, the caspase-3 inhibitor Ac-DEVD-CMK, rapamycin, wortmannin and 7-aminoactinomycin D (7-AAD) were purchased from Sigma-Aldrich (Taufkirchen, Germany). FITC-Annexin-V was bought from BioLegend (San Diego, CA, USA, #640906) and the p38 MAPK inhibitor SB203580 from Calbiochem (San Diego, CA, USA). Recombinant CD95/FasL was kindly provided by Pascal Schneider, Lausanne, Switzerland.

### Caspase activity assays

After HSV-1 infection, mock and infected cells were harvested, lysed and 40 μg of proteins cell lysates were incubated with Ac-DEVD-AMC or Ac-IETD-AFC substrates for the detection of caspase-3 or caspase-8 activity, respectively. Cleavage of the fluorogenic peptide was measured using the Tecan Infinite 200 plate reader at 380/460 nm for AMC substrates and at 400/505 nm for AFC substrates at every minute interval for 30 min.

### Subcellular fractionation and western blot analysis

To prepare total extracts, pelleted, washed cells were directly lysed in 30–50 µl of lysis buffer (20 mM Tris-HCl, pH 7.5, 150 mM NaCl, 5 mM EDTA, pH 8.0, 5 mM Na-pyrophosphate, 1 mM Na_3_VO_4_, 20 mM NaH_2_PO_4_ pH 7.6, 3 mM ß-glycerophosphate, 10 mM NaF, containing 1% Triton X 100 (Thermo Fisher Scientific, Freiburg, Germany) and a protease inhibitor cocktail (ab65621, Abcam, Cambridge, UK). To prepare crude mitochondria, the cells were pelleted, resuspended in MSH buffer (210 mM mannitol, 70 mM sucrose, 20 mM HEPES, pH 7.5, 1 mM EDTA and complete protease inhibitors) and incubated on ice for 30 min. Afterwards the cells were lysed using a syringe with a 23–27 G needle until 50% of the cells were broken (trypan blue positive). The nuclei were removed by centrifugation at 500 x g and a crude mitochondria (heavy membrane) fraction was obtained by an additional centrifugation step at 13000 x g. The mitochondrial fraction was washed twice in MSH buffer and then resuspended in buffer A containing 1 % SDS. Western blotting of total extracts and mitochondrial fractions were performed as previously reported [28, 61]. Expression of β-actin or α-ATPase (V) were used as total or mitochondrial protein loading controls, respectively. Densitometric analysis of scanned films was performed by NIH ImageJ software (versions 1.46r and 1.53k, Bethesda, MD, USA).

### Viability assays

Viability was quantified by FITC-Annexin-V/7-AAD flow cytometry analysis using a FACS Calibur equipment from Becton Dickinson (Heidelberg, Germany) as described [28]. Data were analyzed with the FlowJo software. In other experiments viability was assessed using a commercial MTS colorimetric kit (Cell Titer 96 Aqueous One Solution, Promega, Madison, WI, USA), according to standard procedures.

### Immunofluorescence and electron microscopy

For microscopy analysis, MEFs were directly grown on glass coverslips and infected with HSV-1. U937 cells were infected with HSV-1 and centrifuged at 1200 rpm for 5 min. The cells were fixed by adding a methanol: acetic acid mixture of 3:1 for 10 min. After aspirating the fixative, the cells were smeared on the slide and air dried for 10 min. The cells were blocked with 5% BSA in PBS and consequently incubated in primary and secondary antibodies and a coverslip was placed on the slide after adding 1 drop of mounting medium (Mountant-permafluor, TA-030-FM, Thermo Fisher Scientific, Freiburg, Germany). HSV-1-gD detection by optical immunofluorescence microscopy was performed as previously described except that Hoechst 33342 (2 µg/ml) was used as nuclear stain [61]. For confocal microscopic analysis, MEFs were fixed with 4% paraformaldehyde and permeabilized with 0.2% Triton-X 100 in PBS. Samples were subjected to double immunofluorescence staining using the rabbit polyclonal antibody against LC3B (1:200) and the mouse monoclonal antibody against HSV-1-gD DL6 (1:200). The primary antibodies were revealed using the Alexa Fluor®488-conjugated anti-rabbit and the Alexa Fluor 555-conjugated anti-mouse secondary antibodies (1:200, Invitrogen, Thermo Fisher Scientific, Freiburg, Germany), respectively. Nuclei were counterstained with Hoechst 33342 (Sigma-Aldrich, Taufkirchen, Germany). Samples were analyzed by using the LEICA TCS SP5 confocal microscope (Leica, Heidelberg, Germany). The quantitative analysis of colocalization of LC3 and HSV-1-gD was performed as previously published [62]. Pearson’s correlation (PC) and colocalization rate (CR) were obtained using the Leica application suite for advanced fluorescence software (Leica Instruments); the mean fluorescence intensity and the mean PCs and CRs were calculated by analyzing a minimum of 10 fields/sample, in a blind fashion. The Mean Colocalization Index (CI) was obtained using the formula: CI = CR × PC. For ultrastructural electron microscopy observation, cells were fixed with 2.5% glutaraldehyde in 0.1 M MPB containing 2% sucrose, and then post-fixed with 1% OsO4 in the same buffer. Cells were then dehydrated in ascending ethanol concentrations and embedded in Spurr epoxy resin (Agar Scientific LTD, Stansted, Essex, UK). Ultrathin sections were stained with uranyl acetate and lead citrate and observed under a Philips CM12 transmission electron microscope (Philips Instruments, Eindhoven, The Netherlands) operating at 80 kV.

### Plaque assay

The plaque assay method was used to determine HSV-1 viral titres. 100 µl of the cell supernatant was collected from infected cells and serially diluted from 10^−1^ to 10^−7^ in 900 µl of DMEM + 1% FCS. Vero cells were seeded in 6 well plates the previous day so that they reach ca. 90% confluency on the day of the experiment. In each well, 400 µl of fresh DMEM + 1% FCS and 100 µl of the respective diluted supernatant was added. After 1 h of adsorption on a shaker at 37 °C, the infection was stopped by removing the medium and topping the cells with a semisolid layer of carboxy methyl cellulose CMC (Sigma-Aldrich, Taufkirchen, Germany). This blocks the spread of the virus allowing the infection of only the surrounding cells. After 48 to 72 h, the cells lyse and plaques are formed, which can be stained with crystal violet dye, counted and plaque forming unit (PFU) determined as follows:

$${{{{{{{\mathrm{PFU}}}}}}}} = \frac{{{{{{{{{\mathrm{plaques}}}}}}}}\,{{{{{{{\mathrm{counted}}}}}}}}\,{{{{{{{\mathrm{divided}}}}}}}}}}{{{{{{{{{\mathrm{Dilution}}}}}}}}\,{{{{{{{\mathrm{factor}}}}}}}}\,{{{{{{{\mathrm{x}}}}}}}}\,{{{{{{{\mathrm{diluted}}}}}}}}\,{{{{{{{\mathrm{volume}}}}}}}}}}$$

### Statistical analysis

Statistical analysis and data presentation was performed using GraphPad Prism (v6) software. Data was assessed using parametric one-way analysis of variance (ANOVA) or two-sample Student’s *t*-test. The statistical significances were calculated using the Bonferroni’s Multiple Comparison methods.

## Supplementary information

Supplemental Material

Pre-authorship agreement

## References

[1] Galluzzi L, Vitale I, Aaronson SA, Abrams JM, Adam D, Agostinis P (2018). Molecular mechanisms of cell death: recommendations of the Nomenclature Committee on Cell Death 2018. Cell Death Differ.

[2] Häcker G (2018). Apoptosis in infection. Microbes Infect.

[3] Neumann S, El Maadidi S, Faletti L, Haun F, Labib S, Scheitman A (2015). How do viruses control mitochondria-mediated apoptosis?. Virus Res.

[4] Galluzzi L, Brenner C, Morselli E, Touat Z, Kroemer G (2008). Viral control of mitochondrial apoptosis. PLoS Pathog.

[5] Guo H, Kaiser WJ, Mocarski ES (2015). Manipulation of apoptosis and necroptosis signaling by herpesviruses. Med Microbiol Immunol.

[6] Yu X, He S (2016). The interplay between human herpes simplex virus infection and the apoptosis and necroptosis cell death pathways. Virol J.

[7] He S, Han J. Manipulation of host cell death pathways by herpes simplex virus. Curr Top Microbiol Immunol. 2020. 10.1007/82_2020_196. 10.1007/82_2020_196 32060646

[8] Goodkin ML, Morton ER, Blaho JA (2004). Herpes simplex virus infection and apoptosis. Int Rev Immunol.

[9] Leopardi R, Roizman B (1996). The herpes simplex virus major regulatory protein ICP4 blocks apoptosis induced by the virus or by hyperthermia. Proc Natl Acad Sci USA.

[10] Aubert M, O’Toole J, Blaho JA (1999). Induction and prevention of apoptosis in human HEp-2 cells by herpes simplex virus type 1. J Virol.

[11] Leopardi R, Van Sant C, Roizman B (1997). The herpes simplex virus 1 protein kinase US3 is required for protection from apoptosis induced by the virus. Proc Natl Acad Sci USA.

[12] Munger J, Roizman B (2001). The US3 protein kinase of herpes simplex virus 1 mediates the posttranslational modification of BAD and prevents BAD-induced programmed cell death in the absence of other viral proteins. Proc Natl Acad Sci USA.

[13] Benetti L, Munger J, Roizman B (2003). The herpes simplex virus 1 US3 protein kinase blocks caspase-dependent double cleavage and activation of the proapoptotic protein BAD. J Virol.

[14] Zhou G, Galvan V, Campadelli-Fiume G, Roizman B (2000). Glycoprotein D or J delivered in trans blocks apoptosis in SK-N-SH cells induced by a herpes simplex virus 1 mutant lacking intact genes expressing both glycoproteins. J Virol.

[15] Zhou G, Avitabile E, Campadelli-Fiume G, Roizman B (2003). The domains of glycoprotein D required to block apoptosis induced by herpes simplex virus 1 are largely distinct from those involved in cell-cell fusion and binding to nectin1. J Virol.

[16] Jerome KR, Fox R, Chen Z, Sears AE, Lee H, Corey L (1999). Herpes simplex virus inhibits apoptosis through the action of two genes, Us5 and Us3. J Virol.

[17] Perng GC, Jones C, Ciacci-Zanella J, Stone M, Henderson G, Yukht A (2000). Virus-induced neuronal apoptosis blocked by the herpes simplex virus latency-associated transcript. Science.

[18] Ahmed M, Lock M, Miller CG, Fraser NW (2002). Regions of the herpes simplex virus type 1 latency-associated transcript that protect cells from apoptosis in vitro and protect neuronal cells in vivo. J Virol.

[19] Branco FJ, Fraser NW (2005). Herpes simplex virus type 1 latency-associated transcript expression protects trigeminal ganglion neurons from apoptosis. J Virol.

[20] Sanfilippo CM, Blaho JA (2006). ICP0 gene expression is a herpes simplex virus type 1 apoptotic trigger. J Virol.

[21] Kim JA, Kim JC, Min JS, Kang I, Oh J, Ahn JK (2017). HSV-1 ICP27 induces apoptosis by promoting Bax translocation to mitochondria through interacting with 14-3-3theta. BMB Rep.

[22] Mastino A, Sciortino MT, Medici MA, Perri D, Ammendolia MG, Grelli S (1997). Herpes simplex virus 2 causes apoptotic infection in monocytoid cells. Cell Death Differ.

[23] Ito M, Koide W, Watanabe M, Kamiya H, Sakurai M (1997). Apoptosis of cord blood T lymphocytes by herpes simplex virus type 1. J Gen Virol.

[24] Fleck M, Mountz JD, Hsu HC, Wu J, Edwards CK, Kern ER (1999). Herpes simplex virus type 2 infection induced apoptosis in peritoneal macrophages independent of Fas and tumor necrosis factor-receptor signaling. Viral Immunol.

[25] Pollara G, Speidel K, Samady L, Rajpopat M, McGrath Y, Ledermann J (2003). Herpes simplex virus infection of dendritic cells: balance among activation, inhibition, and immunity. J Infect Dis.

[26] Kather A, Raftery MJ, Devi-Rao G, Lippmann J, Giese T, Sandri-Goldin RM (2010). Herpes simplex virus type 1 (HSV-1)-induced apoptosis in human dendritic cells as a result of downregulation of cellular FLICE-inhibitory protein and reduced expression of HSV-1 antiapoptotic latency-associated transcript sequences. J Virol.

[27] Vanden Oever MJ, Han JY (2010). Caspase 9 is essential for herpes simplex virus type 2-induced apoptosis in T cells. J Virol.

[28] Papaianni E, El Maadidi S, Schejtman A, Neumann S, Maurer U, Marino-Merlo F (2015). Phylogenetically distant viruses use the same BH3-only protein puma to trigger Bax/Bak-dependent apoptosis of infected mouse and human cells. PLoS ONE.

[29] Langelier Y, Bergeron S, Chabaud S, Lippens J, Guilbault C, Sasseville AM (2002). The R1 subunit of herpes simplex virus ribonucleotide reductase protects cells against apoptosis at, or upstream of, caspase-8 activation. J Gen Virol.

[30] Esaki S, Goshima F, Katsumi S, Watanabe D, Ozaki N, Murakami S (2010). Apoptosis induction after herpes simplex virus infection differs according to cell type in vivo. Arch Virol.

[31] Dufour F, Bertrand L, Pearson A, Grandvaux N, Langelier Y (2011). The ribonucleotide reductase R1 subunits of herpes simplex virus 1 and 2 protect cells against poly(I. C)-induced apoptosis. J Virol.

[32] Li H, Wang P, Yu J, Zhang L (2011). Cleaving Beclin 1 to suppress autophagy in chemotherapy-induced apoptosis. Autophagy.

[33] Li H, Wang P, Sun Q, Ding WX, Xin XM, Sobol RW (2011). Following cytochrome c release, autophagy is inhibited during chemotherapy-induced apoptosis by caspase 8-mediated cleavage of Beclin 1. Cancer Res.

[34] Kang R, Zeh HJ, Lotze MT, Tang D (2011). The Beclin 1 network regulates autophagy and apoptosis. Cell Death Differ.

[35] Bjørkøy G, Lamark T, Brech A, Outzen H, Perander M, Øvervatn A (2005). p62/SQSTM1 forms protein aggregates degraded by autophagy and has a protective effect on huntingtin-induced cell death. J Cell Biol.

[36] Pankiv S, Clausen TH, Lamark T, Brech A, Bruun J-AA, Outzen H (2007). p62/SQSTM1 binds directly to Atg8/LC3 to facilitate degradation of ubiquitinated protein aggregates by autophagy. J Biol Chem.

[37] McFarlane S, Aitken J, Sutherland JS, Nicholl MJ, Preston VG, Preston CM (2011). Early induction of autophagy in human fibroblasts after infection with human cytomegalovirus or herpes simplex virus 1. J Virol.

[38] Choi CY, Vo MT, Nicholas J, Choi YB (2022). Autophagy-competent mitochondrial translation elongation factor TUFM inhibits caspase-8-mediated apoptosis. Cell Death Differ.

[39] El Maadidi S, Faletti L, Berg B, Wenzl C, Wieland K, Chen ZJ (2014). A novel mitochondrial MAVS/caspase-8 platform links RNA virus-induced innate antiviral signaling to Bax/Bak-independent apoptosis. J Immunol.

[40] Schrantz N, Bourgeade MF, Mouhamad S, Leca G, Sharma S, Vazaquez A (2001). p38-mediated regualtion of an Fas-associated death domain protein-independent pathway leading to caspase-8 activation during TGFbeta-induced apoptosis in human Burkitt lymphoma B cells BL41. Mol Biol Cell.

[41] Kantari C, Walczak H (2011). Caspase-8 and bid: caught in the act between death receptors and mitochondria. Biochim Biophys Acta.

[42] Young MM, Takahashi Y, Khan O, Park S, Hori T, Yun J (2012). Autophagosomal membrane serves as platform for intracellular death-inducing signaling complex (iDISC)-mediated caspase-8 activation and apoptosis. J Biol Chem.

[43] Huang S, Okamoto K, Yu C, Sinicrope FA (2013). p62/sequestosome-1 up-regulation promotes ABT-263-induced caspase-8 aggregation/activation on the autophagosome. J Biol Chem.

[44] You M, Savaraj N, Kuo MT, Wangpaichitr M, Varona-Santos J, Wu C (2013). TRAIL induces autophagic protein cleavage through caspase activation in melanoma cell lines under arginine deprivation. Mol Cell Biochem.

[45] Oral O, Oz-Arslan D, Itah Z, Naghavi A, Deveci R, Karacali S (2012). Cleavage of Atg3 protein by caspase-8 regulates autophagy during receptor-activated cell death. Apoptosis.

[46] Musarra-Pizzo M, Pennisi R, Lombardo D, Velletri T, Sciortino T (2022). Direct cleavage of caspase-8 by herpes simplex virus 1 tegument protein US11. Sci Rep.

[47] Wirawan E, Vande Walle L, Kersse K, Cornelis S, Claerhout S, Vanoverberghe I (2010). Caspase-mediated cleavage of Beclin-1 inactivates Beclin-1-induced autophagy and enhances apoptosis by promoting the release of proapoptotic factors from mitochondria. Cell Death Dis.

[48] Alexander DE, Ward SL, Mizushima N, Levine B, Leib DA (2007). Analysis of the role of autophagy in replication of herpes simplex virus in cell culture. J Virol.

[49] English L, Chemali M, Duron J, Rondeau C, Laplante A, Gingras D (2009). Autophagy enhances the presentation of endogenous viral antigens on MHC class I molecules during HSV-1 infection. Nat Immunol.

[50] Radtke K, English L, Rondeau C, Leib D, Lippe R, Desjardins M (2013). Inhibition of the host translation shutoff response by herpes simplex virus 1 triggers nuclear envelope-derived autophagy. J Virol.

[51] Siracusano G, Venuti A, Lombardo D, Mastino A, Esclatine A, Sciortino MT (2016). Early activation of MyD88-mediated autophagy sustains HSV-1 replication in human monocytic THP-1 cells. Sci Rep.

[52] Turan A, Grosche L, Krawczyk A, Muhl-Zurbes P, Drassner C, Duthorn A (2019). Autophagic degradation of lamins facilitates the nuclear egress of herpes simplex virus type 1. J Cell Biol.

[53] Orvedahl A, Levine B (2008). Autophagy and viral neurovirulence. Cell Microbiol.

[54] Talloczy Z, Jiang W, Virgin HWT, Leib DA, Scheuner D, Kaufman RJ (2002). Regulation of starvation- and virus-induced autophagy by the eIF2alpha kinase signaling pathway. Proc Natl Acad Sci USA.

[55] Lussignol M, Queval C, Bernet-Camard MF, Cotte-Laffitte J, Beau I, Codogno P (2013). The herpes simplex virus 1 Us11 protein inhibits autophagy through its interaction with the protein kinase PKR. J Virol.

[56] Talloczy Z, Virgin HWT, Levine B (2006). PKR-dependent autophagic degradation of herpes simplex virus type 1. Autophagy.

[57] Yakoub AM, Shukla D (2015). Autophagy stimulation abrogates herpes simplex virus-1 infection. Sci Rep.

[58] Zhao C, Wang M, Cheng A, Yang Q, Wu Y, Zhu D (2018). Programmed cell death: the battlefield between the host and alpha-herpesviruses and a potential avenue for cancer treatment. Oncotarget..

[59] Medici MA, Sciortino MT, Perri D, Amici C, Avitabile E, Ciotti M (2003). Protection by herpes simplex virus glycoprotein D against Fas-mediated apoptosis: role of nuclear factor kappaB. J Biol Chem.

[60] Marino-Merlo F, Papaianni E, Frezza C, Pedatella S, De Nisco M, Macchi B (2019). NF-kappaB-dependent production of ROS and restriction of HSV-1 infection in U937 monocytic cells. Viruses..

[61] Marino-Merlo F, Papaianni E, Medici MA, Macchi B, Grelli S, Mosca C (2016). HSV-1-induced activation of NF-kappaB protects U937 monocytic cells against both virus replication and apoptosis. Cell Death Dis.

[62] Serafino A, Andreola F, Pittaluga E, Krasnowska EK, Nicotera G, Sferrazza G (2015). Thymosin a1 modifies podosome architecture and promptly stimulates the expression of podosomal markers in mature macrophages. Expert Opin Biol Ther.

