# Supplementary material for: Caspase-8 is required for HSV-1-induced apoptosis and promotes effective viral particle release via autophagy inhibition
Source: Cell Death Differ. 2022 Nov 24;30(4):885–96. doi: 10.1038/s41418-022-01084-y (PMC10070401; doi:10.1038/s41418-022-01084-y)
Supplement: Supplementary file 1 — Supplemental Material [file 41418_2022_1084_MOESM1_ESM.pdf]

SUPPLEMENTAL MATERIAL

Uncropped Figures

Fig S1 Caspase-8 is not activated on mitochondria of HSV-1-infected MEFs. Anti-caspase-8 western blot analysis of mitochondrial extracts of MEFs either mock-infected (0 h) or infected with 10 m.o.i. of SFV for 4, 8 and 14 h or with 10 m.o.i. of HSV-1 for 8, 14 and 24 h. No caspase-8 is detected on mitochondria in mock-infected cells. SFV leads to mitochondrial translocation of p55 pro-caspase-8 in 4 h followed by its processing into active p43/p41 and p18 forms within 8-14 h. HSV-1 provokes mitochondrial translocation of pro-caspase-8 only at 24 h without its activation/processing. The α-subunit of mitochondrial ATPase (complex V) served as a loading control.

Fig S2 Caspase-8 activation does not depend on p38 MAPK activation in HSV-1-infected MEFs. Caspase-8 (IETDase) activity assay of total extracts of wt MEFs either mock-infected (0 h) or infected with 10 m.o.i. of HSV-1 for 8, 14 and 24 h in the absence (NT) or presence of 20 μM of the p38 MAPK inhibitor SB203580. Inhibition of p38 MAPK does not affect caspase-8 activation in response to HSV-1 infection. Data represent the means of 3-6 independent experiments ± SD. Statistical evaluation by one-way ANOVA: **p < 0.01; ***p < 0.001.

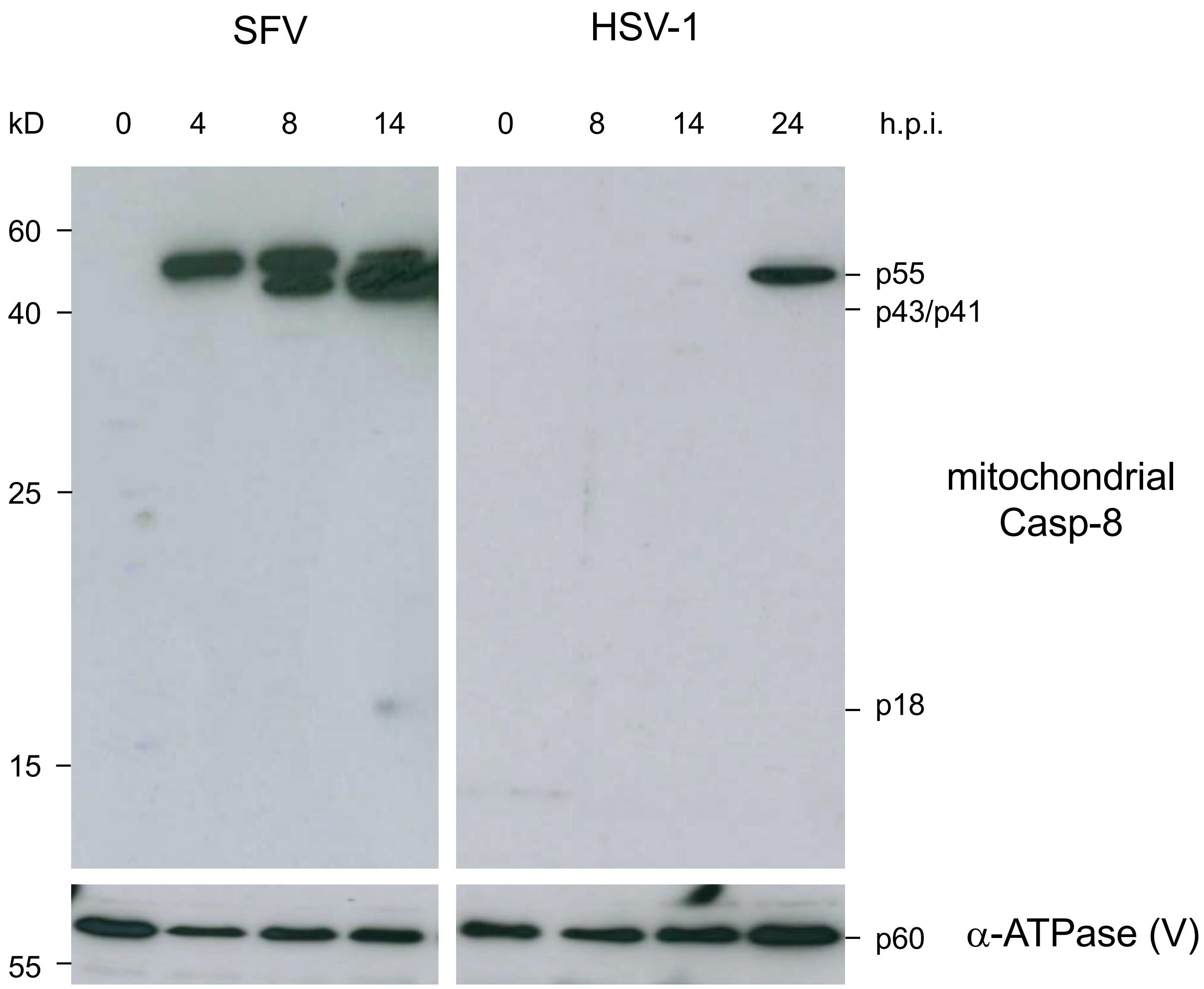
SFV
HSV-1
kD
0
4
8
14
0
8
14
24
h.p.i.
60
40
25
15
55
p55
p43/p41
p18
p60
mitochondrial
Casp-8
α-ATPase (V)

Figure S1

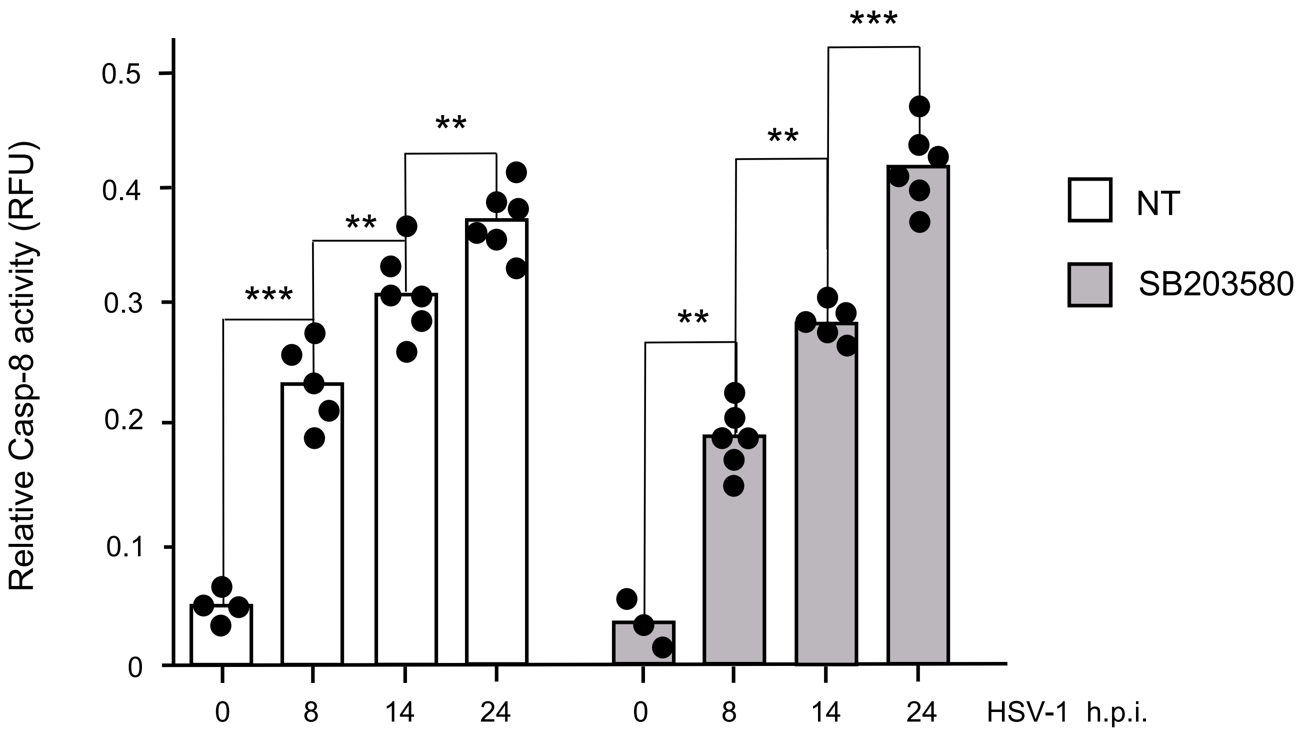
Relative Casp-8 activity (RFU)
0.5
0.4
0.3
0.2
0.1
0
***
**
**
**
**
***
0
8
14
24
0
8
14
24
HSV-1 h.p.i.
NT
SB203580

Figure S2

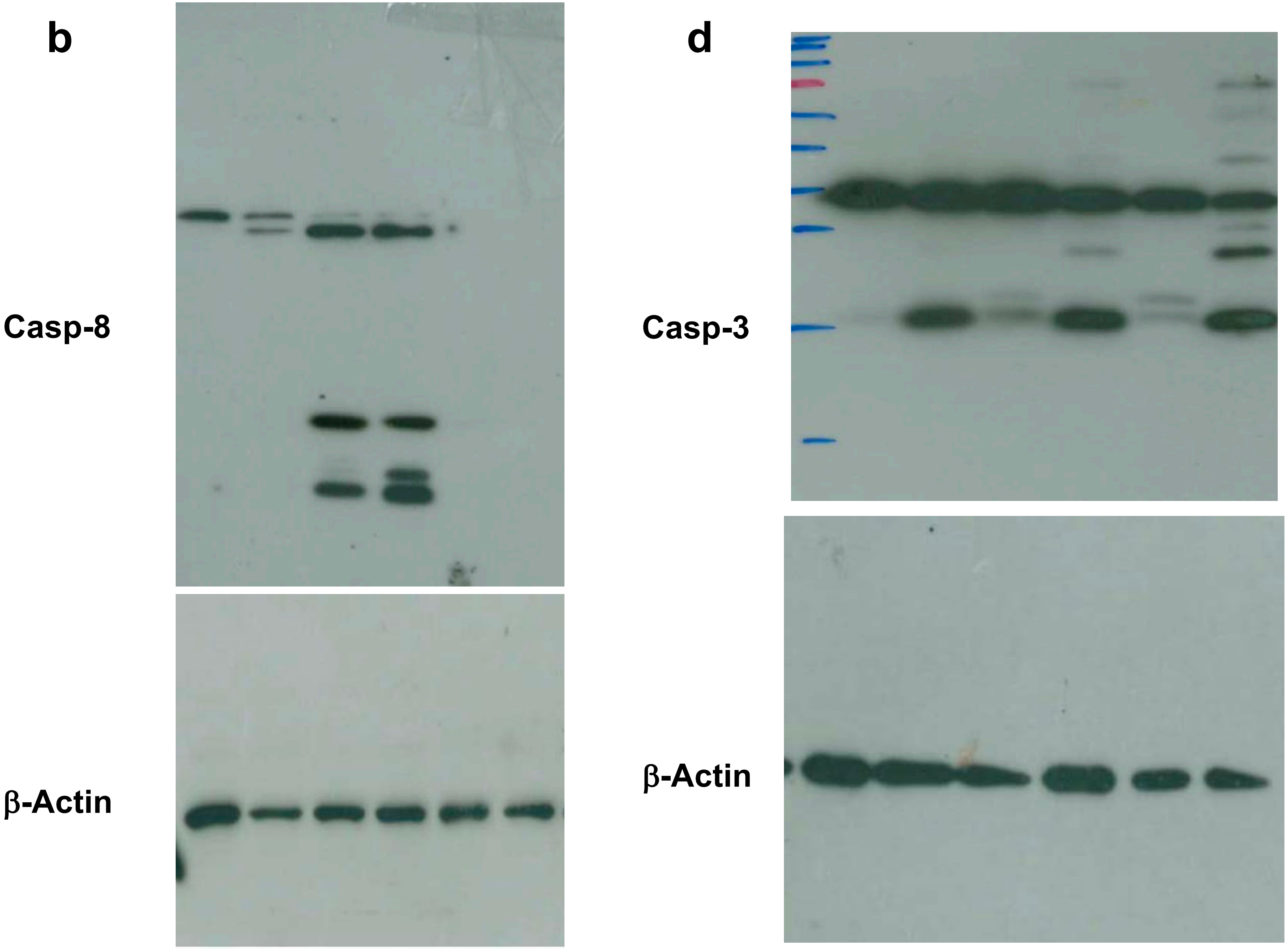

Figure 1

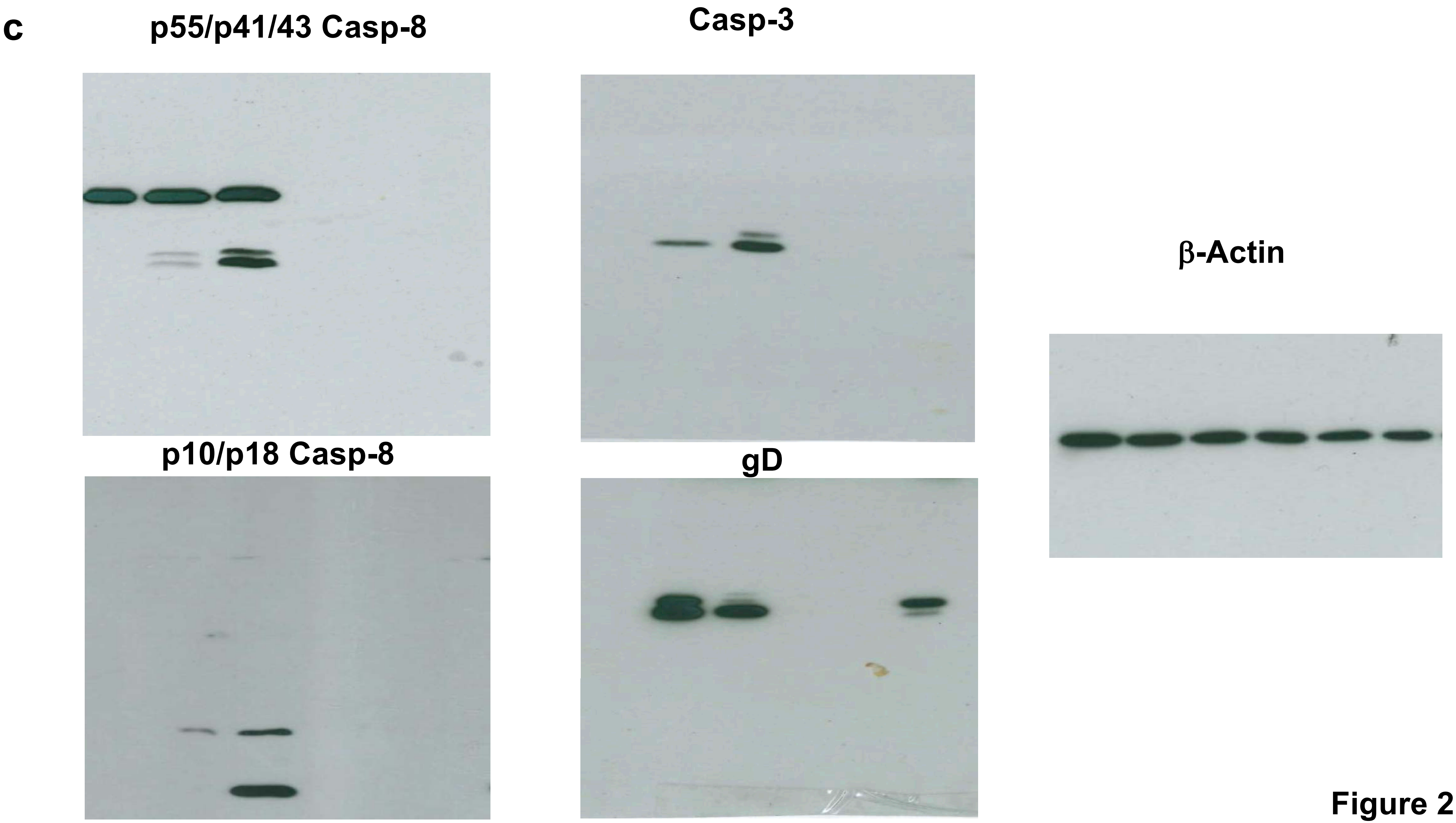

Figure 2

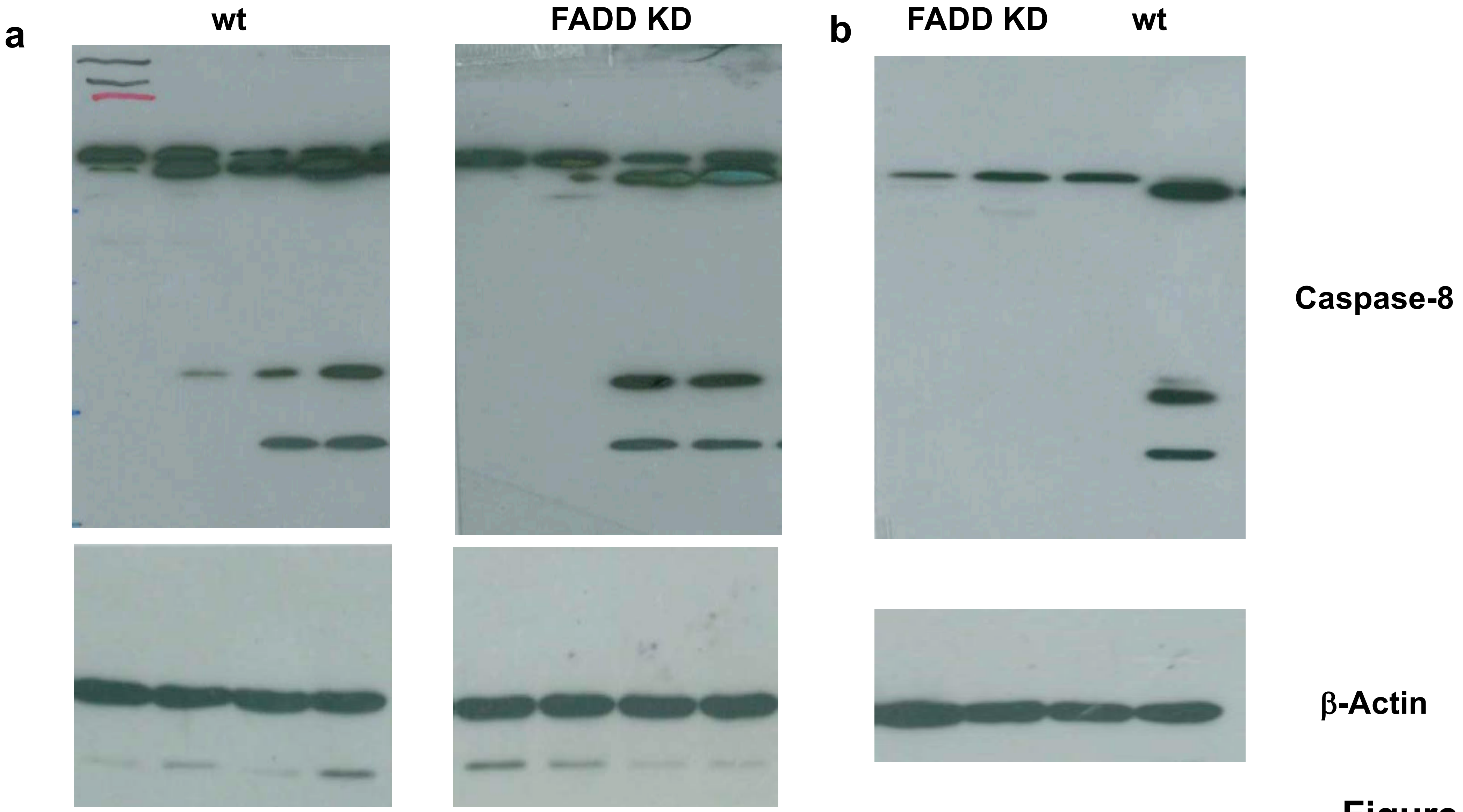

Figure 3

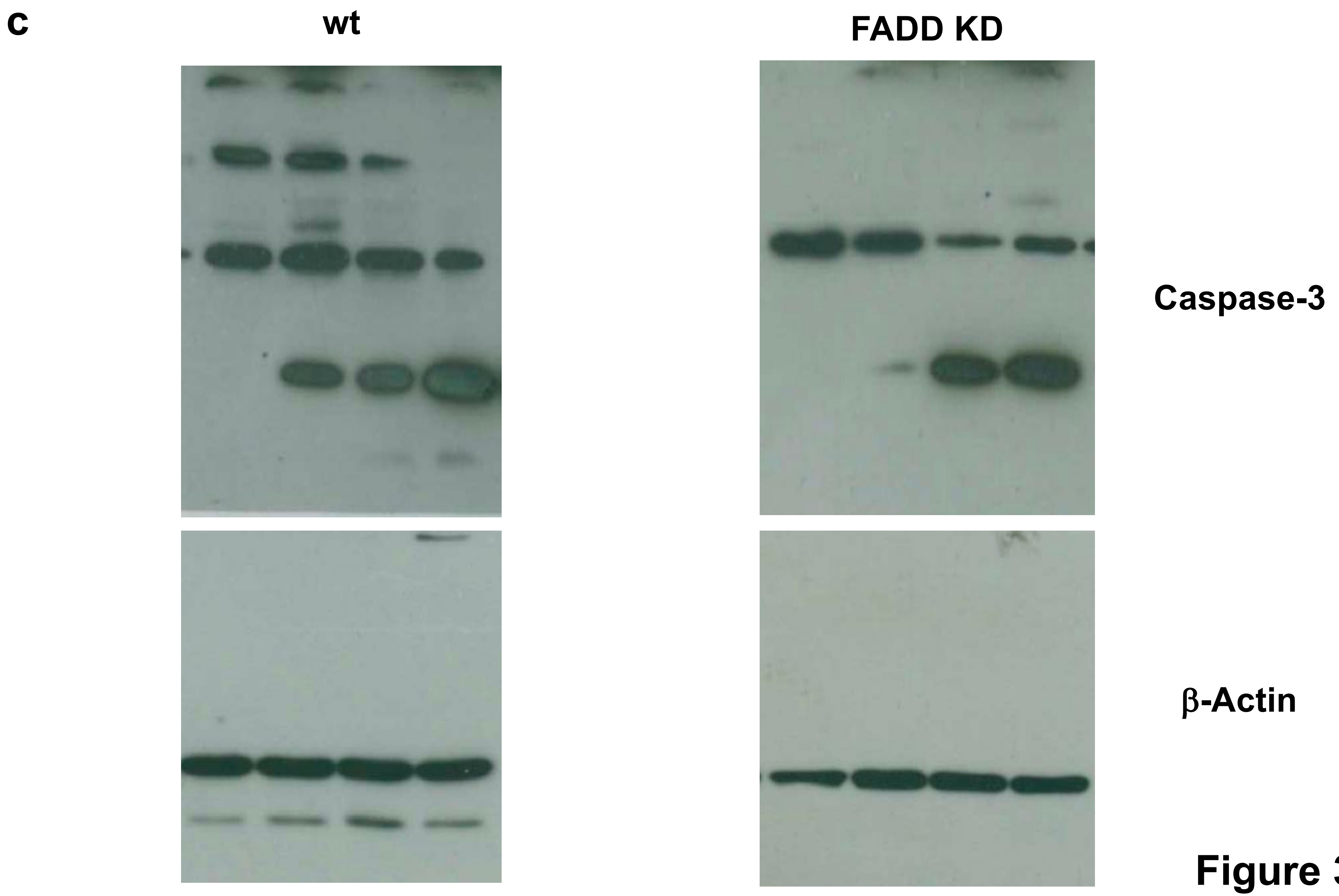

Figure 3

e

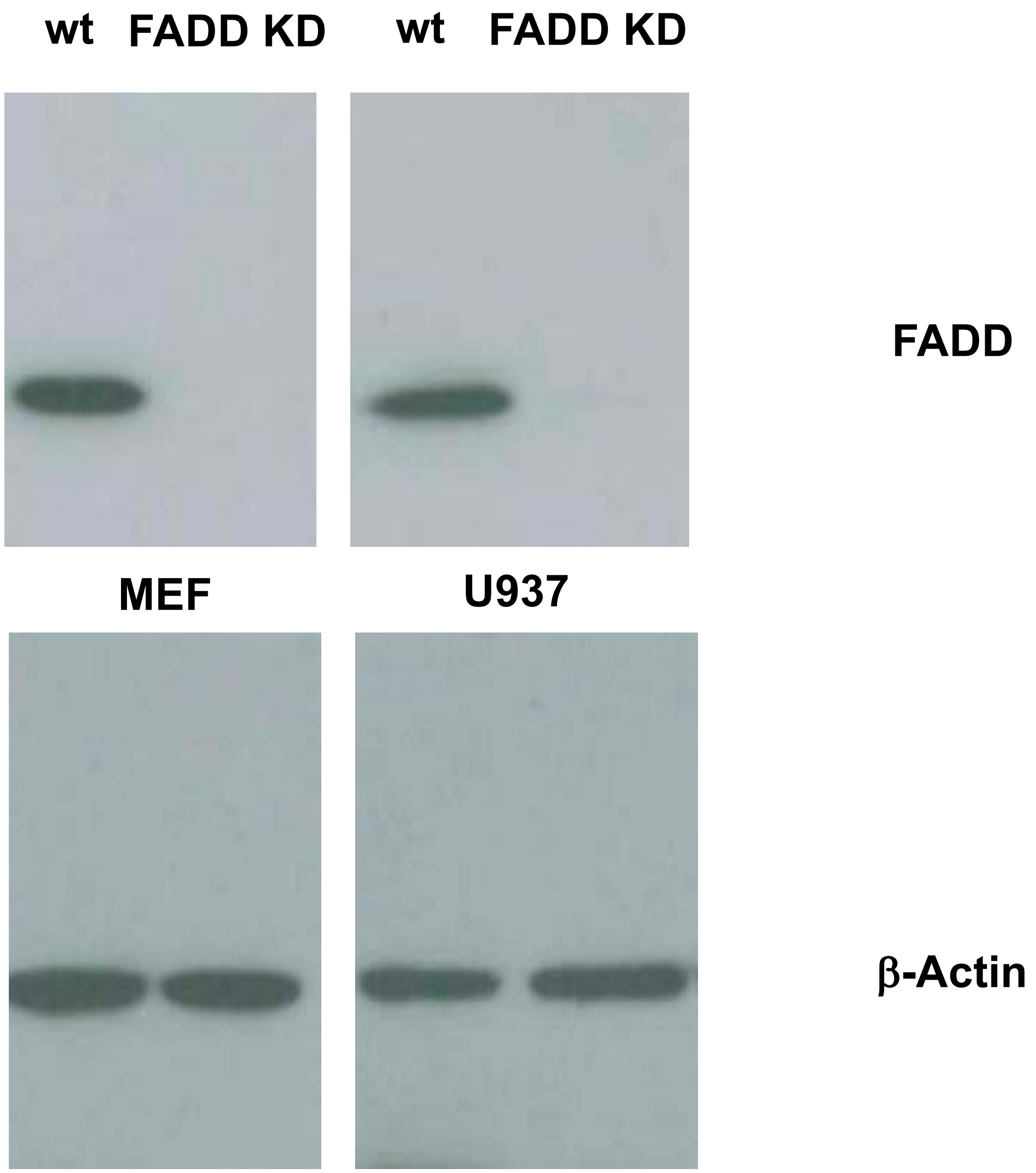

Figure 3

a

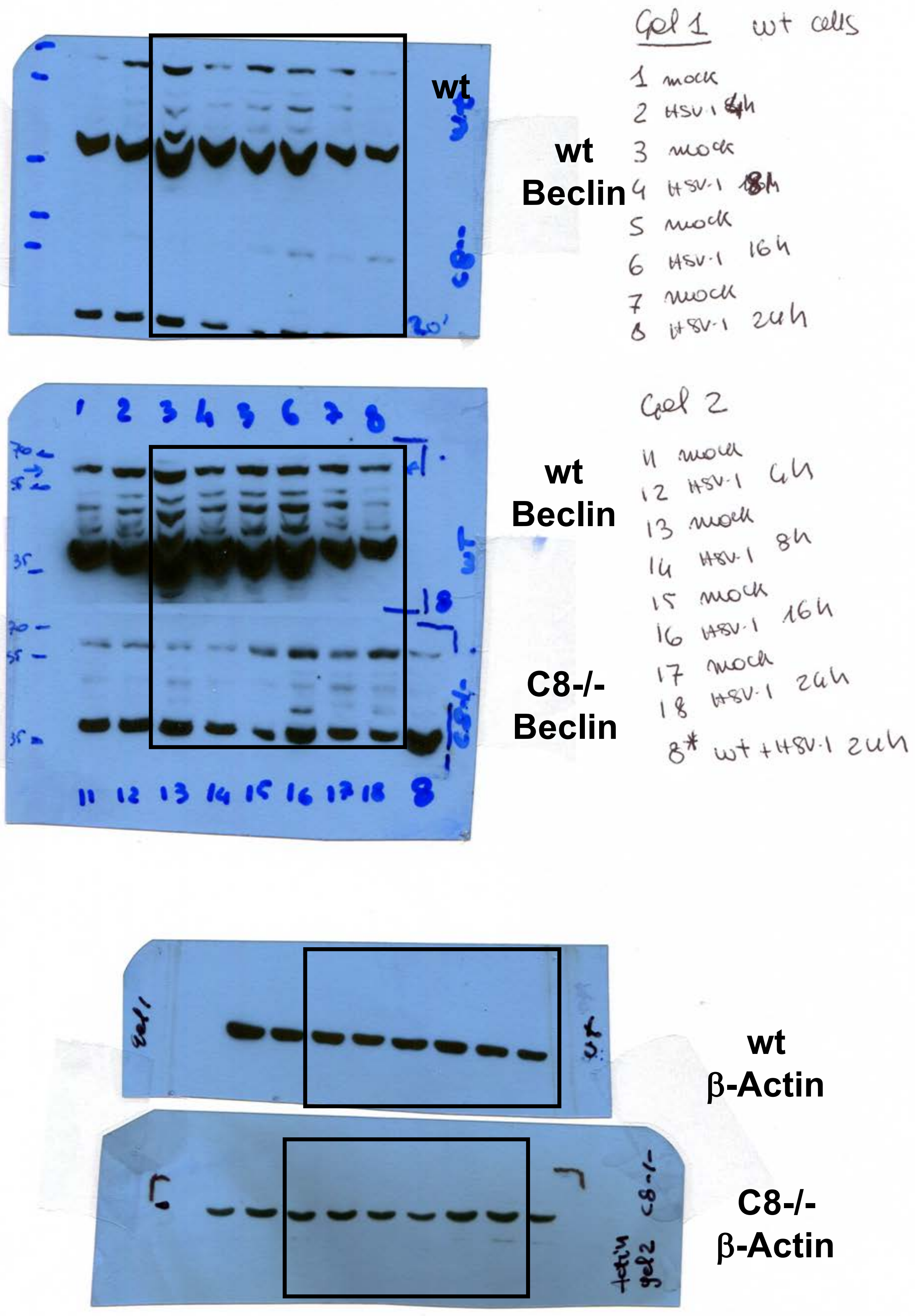
wt
wt
Beclin
wt
Beclin
C8-/-
Beclin
wt
β-Actin
C8-/-
β-Actin
Gel 1 wt cells
1 mock
2 HSV-1 4h
3 mock
4 HSV-1 8h
5 mock
6 HSV-1 16h
7 mock
8 HSV-1 24h
Gel 2
11 mock
12 HSV-1 4h
13 mock
14 HSV-1 8h
15 mock
16 HSV-1 16h
17 mock
18 HSV-1 24h
8* wt + HSV-1 24h
1 2 3 4 5 6 7 8
11 12 13 14 15 16 17 18 8

Figure 5

c

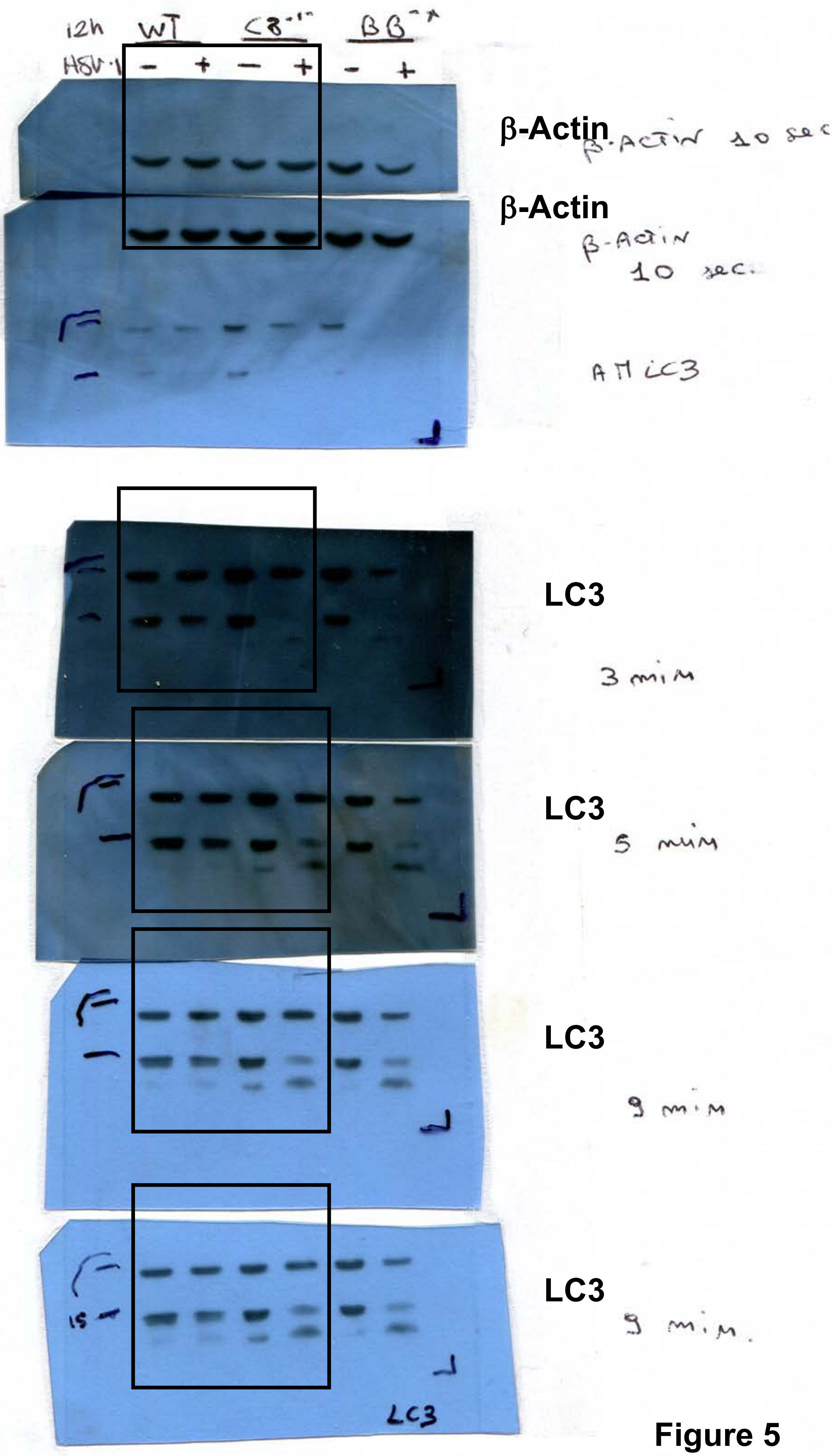
12h
WT
HSV-1
- + - + - +
β-Actin
β-Actin 10 sec
β-Actin
β-Actin 10 sec.
Anti LC3
LC3
3 min
LC3
5 min
LC3
9 min
LC3
9 min.
LC3

Figure 5

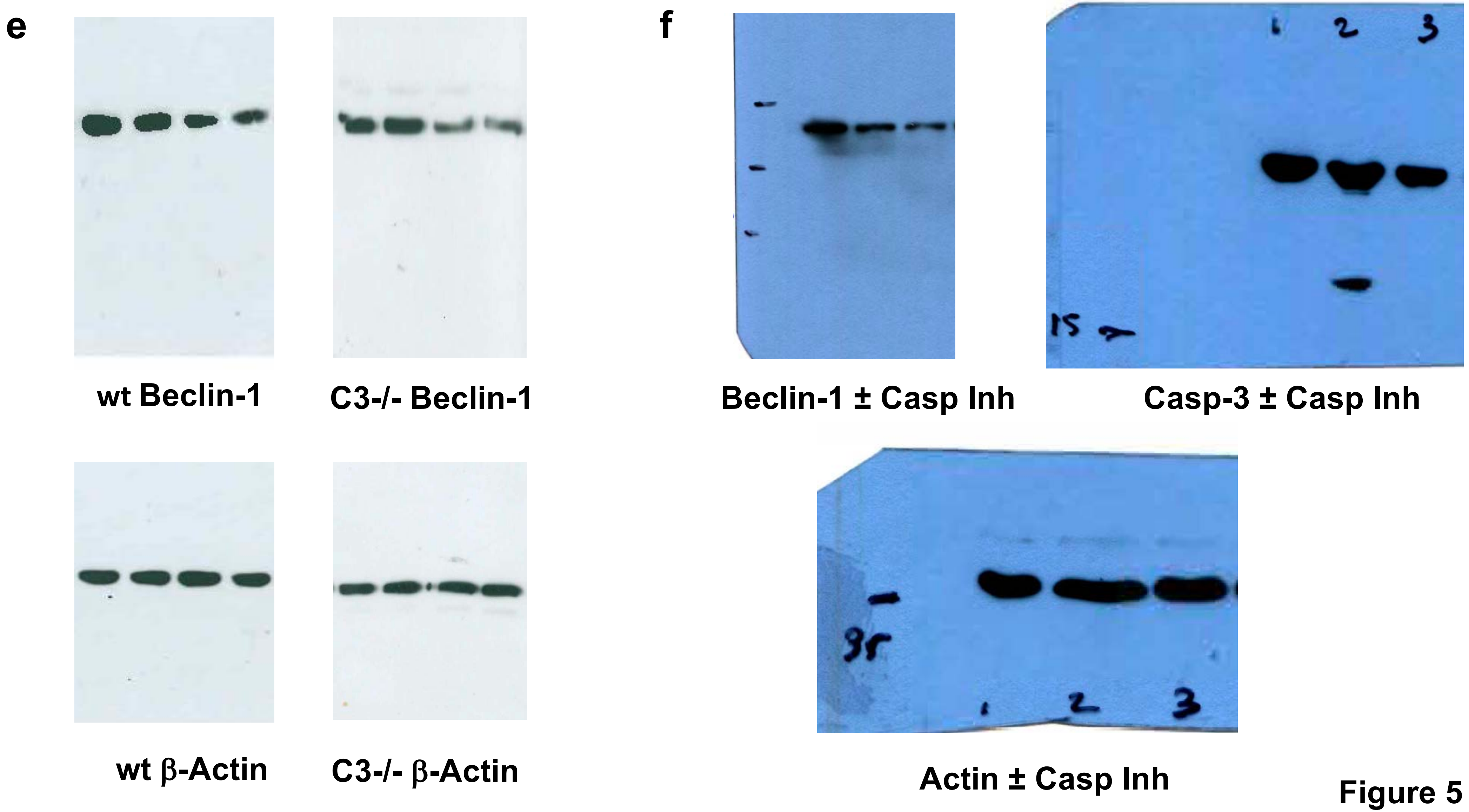
e
wt Beclin-1
C3-/- Beclin-1
wt β-Actin
C3-/- β-Actin
f
Beclin-1 ± Casp Inh
Casp-3 ± Casp Inh
Actin ± Casp Inh

Figure 5

g

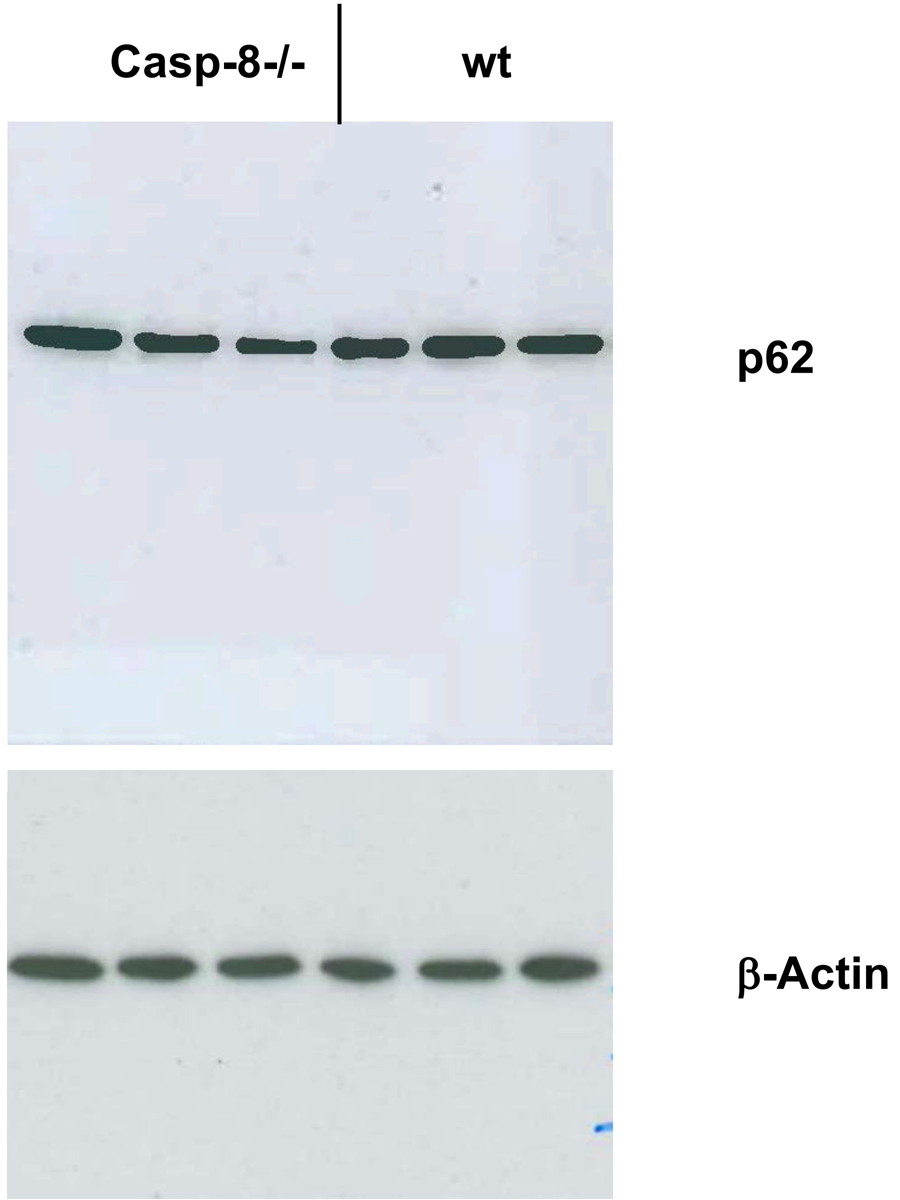

Figure 5

a

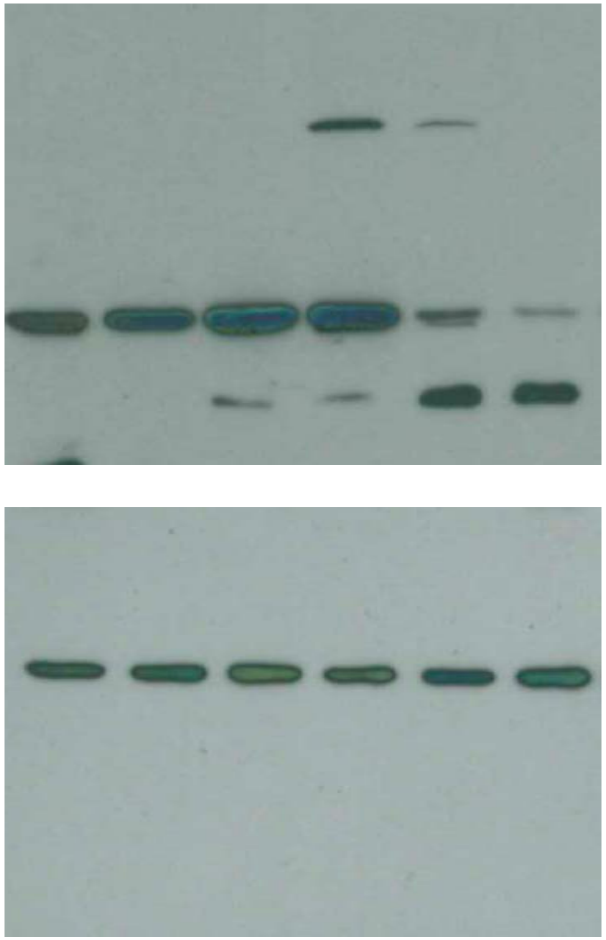

Figure 6

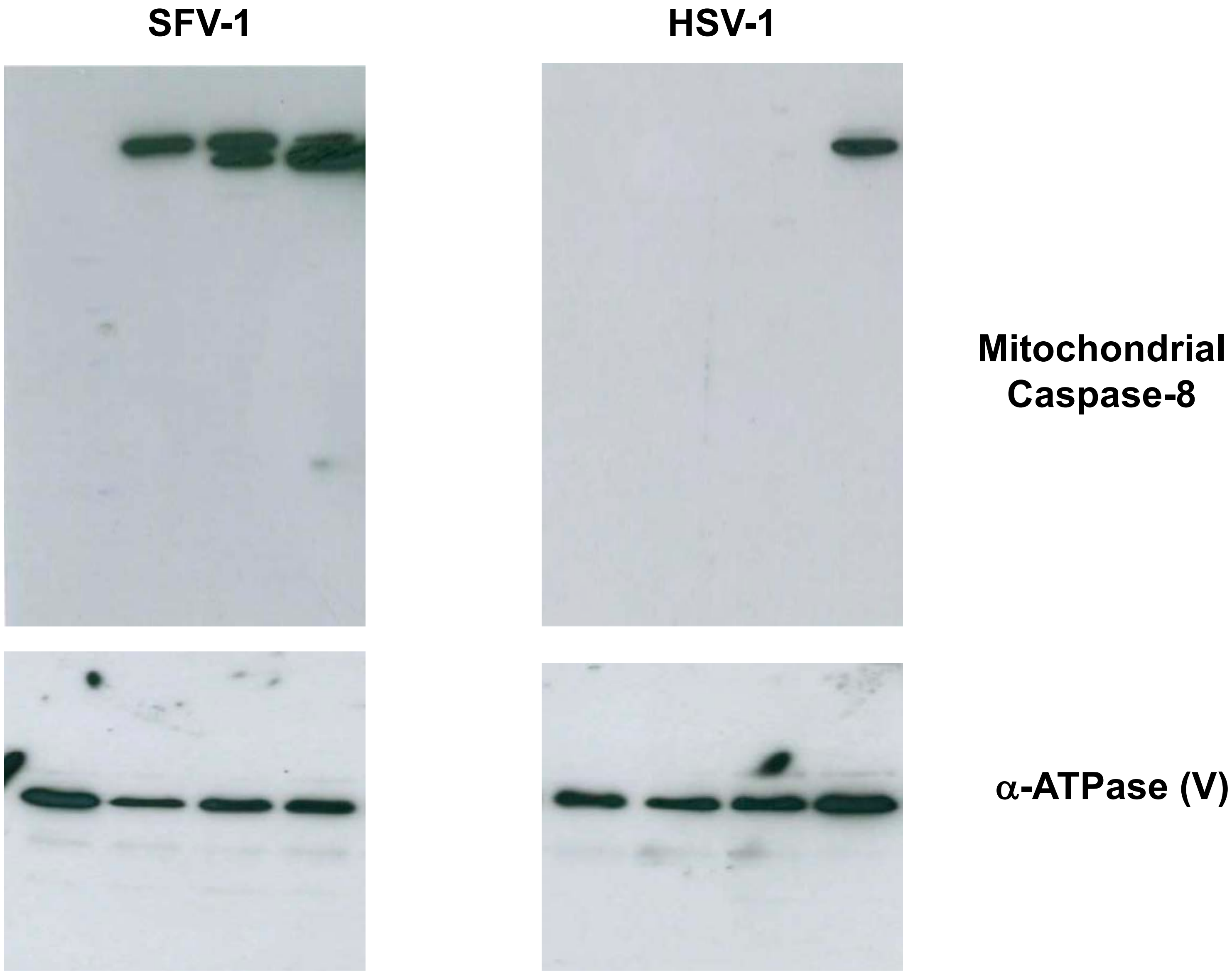

Suppl. Figure 1
