# Supplementary material for: Caspase-8 is required for HSV-1-induced apoptosis and promotes effective viral particle release via autophagy inhibition
Source: Cell Death Differ. 2022 Nov 24;30(4):885–96. doi: 10.1038/s41418-022-01084-y (PMC10070401; doi:10.1038/s41418-022-01084-y)
Supplement: Supplementary file 2 — Pre-authorship agreement [file 41418_2022_1084_MOESM2_ESM.pdf]

SPRINGER NATURE

Change of authorship request form - Journals (pre-acceptance)

Important information. Please read.

- This form should be used by authors to request any change in authorship (adding/deleting authors) including changes in corresponding authors. This form should not be used for name changes. Please fully complete all sections. Use black ink and block capitals and provide each author's full name with the given name first followed by the family name.

- By signing this declaration, all authors guarantee that the order of the authors are in accordance with their scientific contribution, if applicable as different conventions apply per discipline, and that only authors have been added who made a meaningful contribution to the work.

- Please note, in author collaborations where there is formal agreement for representing the collaboration, it is sufficient for the representative or legal guarantor (usually the corresponding author) to complete and sign the Authorship Change Form on behalf of all authors, **next to the added/removed author(s)**. **(Complete Section 3, followed by Section 6.)**
  In author collaborations where there is no formal agreement for representing the collaboration and **there are more than 10 authors**, one may sign for all, provided the signer appends correspondence that attests that each of the authors have agreed to the change **and the added/removed authors sign the form**. **(Complete Section 3, followed by Section 6.)**

- Please note, we cannot investigate or mediate any authorship disputes. If you are unable to obtain agreement from all authors (including those who you wish to be removed) you must refer the matter to your institution(s) for investigation. Please inform us if you need to do this.

- If you are not able to return a fully completed form within **30 days** of the date that it was sent to the author requesting the change, we may have to withdraw your manuscript. We cannot publish manuscripts where authorship has not been agreed by all authors (including those who have been removed).

- Incomplete forms will be rejected.

- Please return/upload this form, fully completed, to the Journals Editorial Office. The Journal and/or Publisher will consider the information you have provided to decide whether to approve the proposed change in authorship. We may decide to contact your institution for more information or undertake a further investigation, if appropriate, before making a final decision.

SPRINGER NATURE

Change of authorship request form - Journals (pre-acceptance)

Section 1: Please provide the current title of manuscript

Manuscript ID no.: CDD-22-0575RR

Title: Caspase-8 is required for HSV-1-induced apoptosis and promotes effective viral particle release via autophagy inhibition

Section 2: Please provide the previous authorship, in the order shown on the manuscript before the changes were introduced. Please indicate the corresponding author by adding (CA) behind the name.

	First name(s)	Family name	ORCID or SCOPUS id, if available
1st author	Francesca	Marino-Merlo	0000-0001-7898-583X
2nd author	Anusha	Klett	
3rd author	Emanuela	Papaianni	0000-0003-4104-3106
4th author	Selene Francesca Anna	Drago	0000-0003-1469-6101
5th author	Beatrice	Macchi	0000-0003-0878-1799
6th author	Maria Gabriela	Rincon	
7th author	Federica	Andreola	Scopus ID: 13007187700
8th author	Annalucia	Serafino	0000-0002-1142-4752
9th author	Ulrich	Maurer	0000-0002-2504-1517
10th author	Sandro	Grelli	0000-0003-1028-3203
11th author	Antonio	Mastino	0000-0002-0338-6450
12th author	Christoph	Borner	0000-0001-5127-7630

SPRINGER NATURE Springer Nature is one of the world’s leading global research, educational and professional publishers, created in May 2015 through the combination of Nature Publishing Group, Palgrave Macmillan, Macmillan Education and Springer Science+Business Media.

SPRINGER NATURE

Change of authorship request form - Journals (pre-acceptance)

Section 3: Please provide a justification for change. Please use this section to explain your reasons for changing the authorship of your manuscript, e.g. what necessitated the change in authorship? Please refer to the (journal) policy pages for more information about authorship. Please explain why omitted authors were not originally included and/or why authors were removed on the submitted manuscript.

Ulrich Maurer wishes to be removed as a co-author of this manuscript because he feels that in the end he has not contributed enough to justfiy a co-authorship.
In the part "CONTRIBUTIONS" we removed the sentence: "UM contributed to the supervision and experimental design."

Section 4: Proposed new authorship. Please provide your new authorship list in the order you would like it to appear on the manuscript. Please indicate the corresponding author by adding (CA) behind the name. <u>If the Corresponding Author has changed, please indicate the reason under section 3</u>.

	First name(s)	Family name (this name will appear in full on the final publication and will be searchable in various abstract and indexing databases)	Affiliated institute	E-mail address
1st author	Francesca	Marino-Merlo	Dept. Chem. Biol., Pharma.Sci, I-Messina	francesca.marino@unime.it
2nd author	Anusha	Klett	Institute of Molecular Medicine, D-Freiburg	anusha.venkatraman@sgbm.uni-freiburg.de
3rd author	Emanuela	Papaianni	Dept. Chem. Biol., Pharma.Sci, I-Messina	emanuelapapaianni@yahoo.it
4th author	Selena Francesca Anna	Drago	Dept. Chem. Biol., Pharma.Sci, I-Messina	selenedrago@hotmail.com
5th author	Beatrice	Macchi	Dept. Chem. Sci. & Tech., I-Rome	macchi@med.uniroma2.it
6th author	Maria Gabriela	Rincon	Institute of Molecular Medicine, D-Freiburg	gabriela.rincon@mol-med.uni-freiburg.de
7th author	Federica	Andreola	Institute Translational Pharma., I-Rome	federica.andreola@ift.cnr.it
8th author	Annalucia	Serafino	Institute Translational Pharma., I-Rome	annalucia.serafino@ift.cnr.it
9th author	Sandro	Grelli	Dept of Exp.Medicine, I-Rome	grelli@med.uniroma2.it
10th author	Antonio	Mastino	Institute Translational Pharma., I-Rome	antonio.mastino@unime.it
11st author	Christoph	Borner	Institute of Molecular Medicine, D-Freiburg	christoph.borner@uniklinik-freiburg.de

---
SPRINGER NATURE Springer Nature is one of the world's leading global research, educational and professional publishers, created in May 2015 through the combination of Nature Publishing Group, Palgrave Macmillan, Macmillan Education and Springer Science+Business Media.

SPRINGER NATURE

Change of authorship request form - Journals (pre-acceptance)

Section 5: Author contribution, Acknowledgement and Disclosures. Please use this section to provide a new disclosure statement and, if appropriate, acknowledge any contributors who have been removed as authors and ensure you state what contribution any new authors made (if applicable per the journal or book (series) policy). Please ensure these are updated in your manuscript - after approval of the change(s) - as our production department will not transfer the information in this form to your manuscript.

New acknowledgements:

We thank Ian Gentle, Freiburg, Germany, for the caspase-8-/- MEFs and Ulrich Maurer, Freiburg, Germany, for kindly providing the LentiCRISPRv2 Luciferase plasmid and for his advice and comments on the manuscript. We are grateful to Pascal Schneider, Lausanne, Switzerland, for generously supplying the recombinant human CD95/FasL, and Andreas Strasser, WEHI, Melbourne, Australia, for the kind gift of the mouse monoclonal anti-FADD antibody.

New Disclosures (financial and non-financial interests, funding):

New Author Contributions statement (if applicable per the journal policy):

AM and CB conceived and supervised the study. FM-M, EP, GR, SFAD, SG, AV, FA, and AS performed the experiments. AM and CB, with the help of FM-M and BM, wrote the manuscript. All authors read and approved the final manuscript.

State ‘Not applicable’ if there are no new authors.

SPRINGER NATURE Springer Nature is one of the world’s leading global research, educational and professional publishers, created in May 2015 through the combination of Nature Publishing Group, Palgrave Macmillan, Macmillan Education and Springer Science+Business Media.

SPRINGER NATURE Change of authorship request form - Journals (pre-acceptance)

Section 6: Declaration of agreement. *All* authors, unchanged, new and removed *must* sign this declaration.
(NB: Please print the form, (docu)-sign and return/upload a scanned copy. Please note that signatures that have been inserted as an image file are acceptable as long as it is handwritten. Typed names in the signature box are unacceptable.) *** Please delete as appropriate. Delete all of the bold if you were on the original authorship list and are remaining as an author.**

	First name	Family name		Signature	Date
1st author	Francesca	Marino-Merlo	I agree to the proposed new authorship shown in section 4		10.11.2022
2nd author	Anusha	Klett	I agree to the proposed new authorship shown in section 4		10.11.2022
3rd author	Emanuela	Papaianni	I agree to the proposed new authorship shown in section 4		10.11.2022
4th authors	Selena Francesca Anna	Drago	I agree to the proposed new authorship shown in section 4		10.11.2022
5th author	Beatrice	Macchi	I agree to the proposed new authorship shown in section 4		10.11.2022
6th author	Maria Gabriela	Rincon	I agree to the proposed new authorship shown in section 4		10.11.2022
7th author	Federica	Andreola	I agree to the proposed new authorship shown in section 4		10.11.2022

SPRINGER NATURE Springer Nature is one of the world's leading global research, educational and professional publishers, created in May 2015 through the combination of Nature Publishing Group, Palgrave Macmillan, Macmillan Education and Springer Science+Business Media.

SPRINGER NATURE

Change of authorship request form - Journals (pre-acceptance)

	First name	Family name		Signature	Date
8th author	Annalucia	Serafino	I agree to the proposed new authorship shown in section 4	Annalucia Serafino	10.11.2022
9th author	Sandro	Grelli	I agree to the proposed new authorship shown in section 4	Sandro Grelli	10.11.2022
10th author	Antonio	Mastino	I agree to the proposed new authorship shown in section 4	Antonio Mastino	10.11.2022
11th author	Christoph	Borner	I agree to the proposed new authorship shown in section 4	[illegible]	10.11.2022
**removed author**	Ulrich	Maurer	I agree to the proposed new authorship shown in section 4 **and the removal*of my name to the authorship list**	[illegible]	10.11.2022

---- End of form ----

SPRINGER NATURE Springer Nature is one of the world's leading global research, educational and professional publishers, created in May 2015 through the combination of Nature Publishing Group, Palgrave Macmillan, Macmillan Education and Springer Science+Business Media.
